# Farnesoid X receptor as marker of osteotropism of breast cancers through its role in the osteomimetism of tumor cells

**DOI:** 10.1186/s12885-020-07106-7

**Published:** 2020-07-10

**Authors:** L. Absil, F. Journé, D. Larsimont, J. J. Body, L. Tafforeau, D. Nonclercq

**Affiliations:** 1 Laboratory of Histology, University of Mons, 6, avenue du Champ de Mars, (Pentagone 1B), B-7000 Mons, Belgium; 2 Laboratory of Human Anatomy and Experimental Oncology, University of Mons, Mons, Belgium; 3 Laboratory of Oncology and Experimental Surgery, Jules Bordet Institute, ULB, Bruxelles, Belgium; 4 Pathology Department, Jules Bordet Institute, ULB, Bruxelles, Belgium; 5 CHU-Brugmann, ULB, Bruxelles, Belgium; 6 Laboratory of Cell Biology, University of Mons, Mons, Belgium

**Keywords:** Bone metastasis, Osteomimetism, Osteotropism, Breast cancer, FXR, Estrogen receptors, ER

## Abstract

**Background:**

The skeleton is the first and most common distant metastatic site for breast cancer. Such metastases complicate cancer management, inducing considerable morbidities and decreasing patient survival. Osteomimetism is part of the complex process of osteotropism of breast cancer cells. Recent data indicate that Farnesoid X Receptor (FXR) is involved in the transformation and progression of breast cancer.

**Methods:**

The expression of FXR, Runt-related transcription factor 2 (RUNX2) and bone proteins were evaluated on two tumor cell lines (MCF-7 and MDA-MB-231) by immunohistochemistry, immunofluorescence and western blotting and quantified.

**Results:**

In a series of 81 breast cancer patients who developed distant metastases, we found a strong correlation between FXR expression in primary breast tumors and the development of bone metastases, especially in patients with histological grade 3 tumors. In in vitro studies, FXR activation by Chenodeoxycholic acid (CDCA) increased the expression of numerous bone proteins. FXR inhibition by lithocholic acid and z-guggulsterone decreased bone protein expression. Short Hairpin RNA (ShRNA) against FXR validated the involvement of FXR in the osteomimetism of breast cancer cells.

**Conclusion:**

Our experimental results point to a relationship between the expression of FXR in breast cancer cells and the propensity of these tumor cells to develop bone metastases. FXR induces the expression of RUNX2 which itself causes the synthesis of bone proteins by tumor cells.

## Background

Breast cancers are the most frequent malignant tumors diagnosed in women of occidental countries. Indeed, one in eight women will develop such cancer during her life [1], and breast cancer is the second cause of cancer death in women [2]. Even though the breast cancer relative survival rate is 91% at 5 years after diagnosis, this tumor is infiltrating or invasive in 80% of cases [3]. Breast cancer is associated with many risk factors including age, environmental factors, early menstruation, late menopause, family history, obesity, alcohol abuse and hormonal replacement therapy after menopause [3, 4]. Metastases complicate breast cancer treatment, mostly due to the organs targeted by the cancer cells.

Bone is frequently invaded by metastatic breast cancer cells. In fact, 65 to 75% of women with advanced breast cancer develop bone metastases [5] and only 20% of these patients with bone metastases survive after 5 years [6]. In addition, bone metastases cause high morbidity and decrease the quality of life for patients by including pain, pathological fracture, impaired mobility, hypercalcaemia and compression of the spinal cord [5, 7]. The calcified bone matrix is rigid but cancer cells can easily invade sinusoids in the bone marrow owing to high blood flow [6, 8]. Several causes are reported to explain the tropism of breast cancer cells to bone such as the microcalcifications in the primary tumor [9], the low pH of the bone matrix associated to osteoclast activity [10], the large panel of growth factors secreted by osteoblasts and osteocytes or by metastatic cells themselves [7, 10], the adhesive molecules expressed by the tumor cells [8, 10] and the expression of proteins involved in osteotropism [RUNX2, receptor activator of nuclear factor kappa-B (RANK), receptor activator of nuclear factor kappa-B ligand (RANKL) and vitamin D receptor] by malignant cells [10, 11].

Estrogen receptor (ER) alpha is a nuclear receptor activated by estrogens (estrone, estradiol and estriol). This receptor is expressed in the endometrium, the ovaries, the testes, the cerebral cortex, the myocardium, the thyroid and the breasts where it is involved in their development. However, this nuclear receptor is associated with breast cancer and is expressed in 75% of cases [12–14]. ER induces an increase of cell proliferation and of cancer progression, decreases apoptosis, transactivates the expression of many proteins like progesterone receptor (used as a marker of ER activity), and can activate different pathways [mitogen-activated protein kinase (MAPK), proto-oncogene tyrosine-protein kinase Src (Src), phosphatidylinositol 3-kinase (PI3K)] [13, 14]. Fortunately, ER-positive breast cancers have a better prognosis, a low grade and are less aggressive [12].

The farnesoid X receptor (FXR) is a member of the ligand-activated transcription factors of the nuclear receptor superfamily [15]. FXR is mainly located in the liver, intestine, adrenal glands and kidneys [16]. It is also lightly expressed in adipose tissue, the heart [17] and blood vessels [18]. This receptor is activated by bile acids and farnesol, a metabolic intermediate in the mevalonate pathway [15]. After activation by its ligand, FXR can bind the ADN FXR response elements as a monomer or as a heterodimer with its common partner, namely the retinoid X receptor (RXR) [19]. FXR is a bile acid sensor and controls bile acid metabolism in particular by regulating its synthesis, transport and absorption. Indeed, a high level of bile acid induces FXR activation and causes a decrease of bile acid synthesis by inhibiting Cytochrome P450 7A1 (CYP7A1), the rate-limiting enzyme in the bile acid synthesis pathway [19, 20]. Furthermore, FXR plays a role in the regulation of diverse genes involved in lipid and glucose metabolisms [15, 16]. However, a decrease or increase of FXR expression can lead to several pathologies, such as gastrointestinal disorders, liver hypertrophy, liver cirrhosis, cholestasis, atherosclerosis, inflammation and cancer [15, 20, 21]. Regarding to cancer, a loss of FXR expression has been associated with tumorigenic phenotypes in the liver, intestine and colon, where FXR has an antitumor function [22, 23], while its overexpression may also induce non-small cell lung cancer (NSCLC) and esophageal adenocarcinoma [15, 16].

High expression of FXR promotes the propensity of breast tumor cells to metastasize, and previous studies have highlighted a positive correlation between FXR expression and the tumoral proliferation rate, evidenced by Ki-67 marker [16, 18]. Moreover, our previous study showed that FXR activation by CDCA increased cell proliferation in MCF-7 cell line but not in MDA-MB-231 one [18]. Furthermore, an increase of bile acids in plasma and cyst fluid has the ability to promote the development of breast cancer [24]. The same study showed that the breast cancer cell line MDA-MB-231 was attracted by lipid extracts isolated from bone [24]. Finally, FXR was found to be expressed in 100% of primary breast cancers with bone metastases, while it was only expressed in 61% of primary breast cancers with visceral metastases [25].

In the present study, we assess FXR expression and its median score in primary breast cancer in connection with the first site of metastases in order to investigate its involvement in the osteotropism of breast cancer. Subsequently, we investigate, in vitro, the mechanisms whereby FXR expression could induce the osteomimetism of the triple negative MDA-MB-231 breast cancer cells and the ER-positive MCF-7 ones. Osteomimetism of breast cancer cells could explain the osteotropism of breast cancer and the bone metastasis. The osteomimetism of breast cancer cells has been studied by targeting a panel of proteins expressed by bone cells including the transcription factor RUNX2 (Runt-related transcription factor), involved in skeletal development, osteoblast proliferation and differentiation, bone matrix synthesis, and the bone matrix proteins osteopontin (OPN), osteocalcin (OC) and bone sialoprotein (BSP) [26, 27].

## Methods

### Breast cancer tissue sampling

Formalin-fixed, paraffin-embedded primary breast cancer samples, collected from 81 patients at the Jules Bordet Institute, were used for the evaluation of FXR expression. Patients with primary invasive breast cancer were included in the study, while patients with bilateral breast cancer, as well as previous or concomitant cancer other than breast cancer were excluded. The study was performed according to the REMARK recommendations from the National Cancer Institute—European Organisation for Research and Treatment of Cancer (EORTC) [28]. The ethics committee of the Institute approved the use of the tissue material for this study.

### Immunohistochemistry and scoring of FXR

Tissue microarrays (TMA) blocks were cut and tissue sections were mounted on poly-l-Lysine-coated glass slides. Immunohistochemical staining was performed with an antibody raised against human FXR (mouse monoclonal anti-human FXR/NR1H4 antibody, clone A9033A, R&D Systems, Minneapolis, MN, USA). Prior to immunostaining, antigen retrieval was achieved by microwave pretreatment (2 × 10 min at a power of 650 W) in pH 6.0 citrate buffer. Thereafter, the tissue sections were incubated for 30 min at 37 °C in presence of the primary antibody diluted to 1:25. The FXR antigen–antibody reaction was visualized using the Ventana automated system with the highly sensitive Nexes reagents (Enhanced Nexes reagent, Ventana Medical Systems, Tucson, AZ, USA). Positivity was defined as nuclear staining. FXR expression was scored from 0 to 8 by adding a score reflecting the proportion of positively stained cells (none: 0; < 1/100: 1; 1/100 to 1/10: 2; 1/10 to 1/3: 3; 1/3 to 2/3: 4; and > 2/3: 5) and a score reflecting the staining intensity (none: 0, weak: 1, intermediate: 2 and strong: 3), as defined by Allred et al. [29]. Semi-quantitative analysis was performed in a single-blind fashion by an experienced pathologist (D.L.).

### Cell culture

Breast cancer cell line (MCF7) was isolated from a 69-year-old Caucasian woman in 1970 and breast cancer cell line (MDA-MB-231) was established from a pleural effusion of a 51-year-old Caucasian women with a metastatic breast adenocarcinoma. Both cell lines were initially obtained from the American Type Culture Collection (ATCC) (Manassas, VA, USA) [ATCC-HTB-26 and ATCC-HTB-22, respectively]. Both cell lines were tested every two months to verify that they were not infected with mycoplasma. Tests for mycoplasma were negative for all duration of the experiment. Cells were cultured at 37 °C with 95% air and 5% CO_2_ in a humidified atmosphere. Tumor cells were cultured in Dulbecco’s Modified Eagle Medium (DMEM) with phenol red and 25 mM Hepes (BioWhittaker®) supplemented with 10% fetal bovine serum (FBS) (Gibco®), 2 mM L-glutamine (Gibco®), 100 U/ml penicillin, 100 μg/ml streptomycin and 0.25 μg/ml amphotericin b (BioWthittaker®). The cells were passed once a week and further cultured in 75-cm^2^ flasks. The medium was changed every 2–3 days. For the passages, the cells were harvested by trypsinization with trypsin (0.1%) and ethylenediaminetetraacetic acid (EDTA) (0.02%) solution. For the experiments, the cells were seeded in steroid-free medium consisting of DMEM without phenol red supplemented with 10% dextran-coated charcoal-treated FBS (PAN-Biotech®) to suppress the potential influence serum-derived lipid compounds.

### Hormones, drugs and primary antibodies

17β-estradiol (E_2_), 4-hydroxytamoxifen, chenodeoxycholic acid (CDCA) and lithocholic acid (LCA) were obtained from Sigma Aldrich®, fulvestrant (ICI 182,780) from Tocris® (Bristol, UK) and Z-guggulsterone from Enzo Life Sciences®. These compounds were first prepared as stock solution at 10^− 2^ M in ethanol 99.8%, except for 4-hydroxytamoxifen and fulvestrant, which were prepared as stock solution at 10^− 3^ M. All preparations were done under sterile conditions.

A murine monoclonal antibody against FXR was purchased from R&D Systems®. Polyclonal rabbit antibodies against osteopontin (anti-OPN) and RUNX2 were purchased from Abcam®. Polyclonal rabbit antibodies against estrogen receptor alpha, osteocalcin (anti-OC) and a mouse monoclonal antibody against bone sialoprotein (anti-BSP) came from Santa Cruz Biotechnologies®. Except for the RUNX2 antibody (dilution 1/100), all primary antibodies were diluted at 1/50 in PBS (pH 7.2) containing casein 0.05% (Sigma Aldricht®) for immunofluorescence staining.

### Immunofluorescence

The cells were seeded in 12-well-dishes on sterile round glass coverslips (40,000 cells/well for 24 h treatment or 20,000 cells/well for 48 h treatment) in steroid-free medium without phenol red, supplemented with 10% dextran-coated charcoal-treated FBS. The cells were cultured for 24 h without any treatment. After that, the cells were exposed to different treatments (10^− 9^ M 17β-estradiol, 10^− 7^ M 4-hydroxytamoxifen, 10^− 7^ M fulvestrant, 2 10^− 5^ M chenodeoxycholic acid, 8 10^− 5^ M lithocholic acid, 4 10^− 5^ M Z-guggulsterone) or vehicle and the cultures were stopped after 24 h in order to evidence FXR and RUNX2, or after 48 h to detect OPN, OC and BSP. The cells were fixed with paraformaldehyde 4% for 20 min. at 4 °C and processed for immunofluorescence, as detailed in a previous publication [30]. In short, the cells were permeabilized in PBS/Trion X-100 0.1% for 15 min. and incubated in a blocking solution (PBS with casein 0.05%) for 15 min. Primary antibodies were diluted in casein 0.05% and incubated overnight at 4 °C. This step was followed by a 30 min. Exposure to anti-rabbit/mouse peroxidase complexes (ImmPress™ Reagent Kit, Vector Laboratories®), a rabbit anti-peroxidase (1/300 in casein 0.05%) and an anti-rabbit biotinylated antibody (1/50 in casein 0.05%, Vector Laboratories). The cells were rinsed three times for 5 min. With PBS/Triton X-100 0.1% between each incubation with antibodies. Finally, the cell cultures were incubated with Texas Red/Streptavidin (1/50 in PBS, Vector Laboratories®) for 30 min. and rinsed once in PBS and twice in distilled water for 5 min. Each. The preparations were mounted in Vectashield mounting medium containing 4′,6-diamidino-2-phenylindole (DAPI) (Vector Laboratories®). The cell preparations were examined on a Leitz® Orthoplan microscope equipped with a Ploem system for epi-illumination. An excitation wavelength of 560 nm and emission wavelength of 590 nm were used for the observation of Texas red fluorescence. Photos were taken using a high sensitivity camera (Leica® DFT7000 T, Germany) and images were recorded using specialized software (Leica Application Suite X, LAS X, Germany). The cultures were observed at 250x magnification, each microscopic field representing an area of 145,200 μm^2^. Nine fields were taken at random on three independent cultures for each condition of treatments and controls.

### Quantification of fluorescence

The intensity of the fluorescent signal recorded in different experimental groups was evaluated using image processing software (KS 400 imaging software, Carl Zeiss Vision®, Hallbergmoos, Germany). Bone protein immunofluorescence intensity measures were done on nine fields picked at random on three independent cultures for each treatment and for controls. Immunofluorescence intensity measures for dimers ER-FXR were done on four independent cultures for each processing time and controls. For each treatment, bone protein expressions in cell cultures, the mean immunofluorescent signal ± SD was calculated and presented in histograms. The same method of quantification was used with images obtained from proximity ligation assay (PLA). This last method was used to detect direct interaction between ER and FXR as detailed in a following paragraph of the Material and Methods.

### Gene silencing with short hairpin RNA (shRNA)

The shRNA targeting the human FXR was synthesized by Origene (Seraing, Belgium). The shRNA negative control (scramble) from Origene was used to evaluate the nonspecific effects on gene expression. The Short Hairpin RNA (shRNA) is included in pGFP-V-RS vector with puromycin as antibiotic selection under the control of SV40 promoter. MCF-7 cells (2 × 10^5^/well, 6-well-dishes) and MDA-MB-231 cells (1.5 × 10^5^/well, 6-well-dishes) were cultured in DMEM for 24 h and transfected for 48 h with 1 μg DNA plasmid using jetPrime® transfection reagent kit according to the manufacturer’s instructions (Polyplus transfection, France). Transfected cells were fed fresh DMEM with puromycin (0.5 μg/ml for MCF-7 and 1 μg/ml for MDA-MB-231) and further cultured for 7 days. Clones were isolated and cultured to extract proteins dedicated to Western blot analysis or to perform protein detections by immunofluorescence.

### Western blot analysis

Cells were plated in 6 well-dishes (5 × 10^5^/well) containing DMEM. Cell monolayers were harvested and lysed using Radio-Immuno-Precipitation-Assay (RIPA) buffer. Protein concentrations were determined by the Bradford Protein Assay (Roth, Germany) on water-diluted protein samples using Bovine Gamma Globulin as standard. Equal amounts of cell proteins or 25 ng of recombinant FXR protein were subjected to Western blotting using a rabbit polyclonal anti-human FXR/NR1H4 antibody (Abcam, Cambridge, UK) diluted 1/1000. β-actin was used as a loading control, and was detected with a mouse monoclonal anti-human β-actin antibody (SantaCruz, Dallas, USA) diluted 1/500. Peroxidase-labeled anti-rabbit IgG antibody (Invitrogen, 1/1000) or peroxidase-labeled anti-mouse IgG antibody (1:10,000) (Invitrogen, USA) was used as secondary reagents to detect corresponding primary antibodies. Bound peroxidase activity was revealed using the SuperSignal® West Femto Chemiluminescent Substrate (ThermoFisherScientific, USA). Immunostaining signals were digitalized with MY ECL Imager (ThermoScientific, USA). Immunoreactive band intensities were quantified using the software ImageJ®.

### Proximity ligation assay (PLA)

Direct interaction between ER and FXR was assessed by proximity ligation assay (PLA). Cells were seeded in 12-well-dishes (4 × 10^4^/well) on sterile round glass coverslips in steroid-free medium without phenol red, supplemented with 10% dextran-coated charcoal-treated FBS and cultured for 24 h. The cells were then exposed to different treatments for 30 s to 1 h. The cells were then fixed with paraformaldehyde 4% for 20 min at 4 °C and permeabilized in PBS/Triton X-100 0.1% for 15 min and incubated in blocking solution (PBS with casein 0.05%) for 15 min. Both primary antibodies, anti-FXR and anti-ER, were diluted at 1/50 in a solution of PBS with casein 0.05% and incubated overnight at 4 °C. This was followed by 1 h exposure at 37 °C to Duolink PLA probe MINUS and Duolink PLA probe PLUS (1/5 in antibody diluent, Olink, Sigma Aldrich®). The cells were then incubated for 30 min at 37 °C with Duolink Ligation stock and ligase (1/5 and 1/40 respectively in miliQ water, Olink, Sigma Aldrich®). The cells were rinsed twice for 5 min with buffer A (Olink, Sigma Aldrich®) between each stage, and were then incubated for 100 min at 37 °C in dark conditions with Duolink Amplification stock and polymerase (1/5 and 1/80 respectively in miliQ water, Olink, Sigma Aldrich®). Finally, the cells were rinsed twice for 10 min with buffer B (Olink, Sigma Aldrich®) and once with buffer B 1%, and the preparations were mounted in mounting medium with DAPI (Olink, Sigma Aldrith®). The cell preparations were examined with confocal microscopy using an Olympus FV1000D laser scanning inverted microscope equipped with a red laser diode (LD559).

### Statistical analysis

SigmaPlot® 11 software was used for the statistical analysis. The non-parametric Mann-Whitney test was used for the clinical morphological study performed on tumoral biopsies. Parametric analyses were achieved for in vitro studies. Student’s *t* test was performed for the comparison of two groups and One-Way ANOVA for more than two groups, followed by Dunnet’s post-hoc test. For all experiments, *p* values < 0.05 were considered as significant.

## Results

### Expression of FXR in breast cancer tissue

**Fig. 1:**
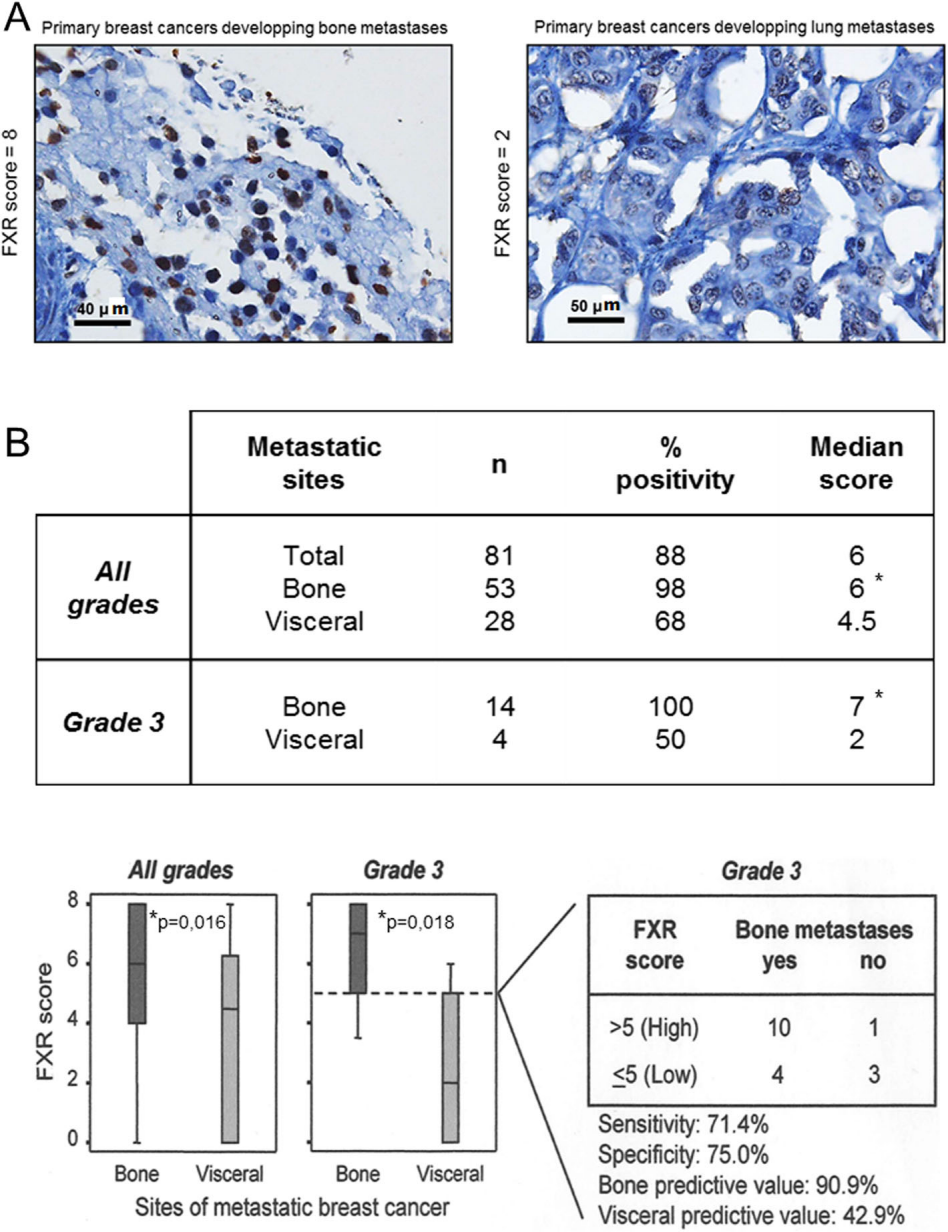
Panel **a** FXR expression in a case of breast cancer with bone metastases (score of 8) and a case with lung metastases (score of 2). The FXR score is higher in bone metastases (8) compared to lung metastases (2). Scale bars = 40 and 50 μm Panel **b** FXR expression in primary tumors in relation with first metastatic site. FXR was expressed in 98% of primary breast cancer with bone metastasis (median score 6) and in 68% (median score 4.5) with visceral metastasis. In grade 3 cancers, the predictive value of FXR was improved. The bone predictive value was 91% and the visceral one was 43%

The potential implication of FXR in the osteotropism of tumoral cells derived from a primary breast tumor, was tested by immunohistochemical detection of FXR in primary tumors and correlated to the first site of metastases. FXR-immunoreactivity was evidenced in nuclei of tumoral cells and we found a higher expression in bone metastases compared to visceral metastases (Fig. 1-a). FXR-immunolabeling was detected in 98% of breast cancer tissue samples (n = 53; median score:6) derived from patients who have developed bone metastases. By contrast, it was detected only in 68% of breast cancer tissue samples (n = 28; median score:4.5) of patients showing visceral metastases (Fig. 1-b). Moreover, the sub-cohort of patients with histological grade 3 tumors and who developed bone metastases presented a strong FXR-expression (median score = 7) in all samples. By contrast a lower incidence (50% of samples) and a lower FXR-expression score (median score = 2) iwas found in patients who developed visceral metastases. On the basis of a cutoff score of 5 for FXR expression, we observe a strong correlation between the strong level of FXR immunoreactivity in the primary tumors and the development of bone metastases (high predictive value of 91%) (Fig. 1-b). These clinical results point to an involvement of FXR in the osteotropism of breast cancer.

### FXR expression in breast cancer cells

**Fig. 2:**
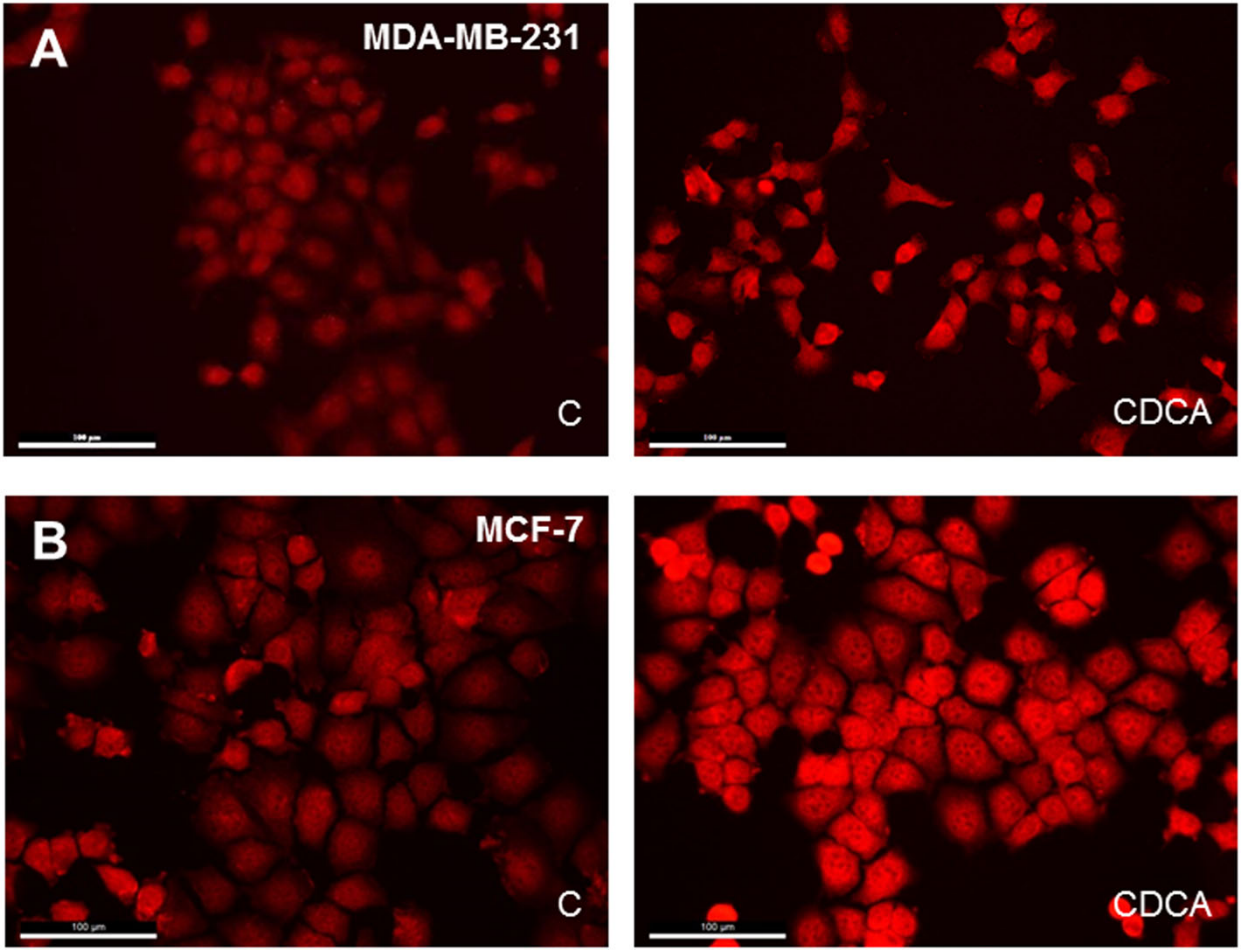
FXR detection by immunofluorescence in control culture (C) and after a CDCA treatment for 24 h (CDCA). **a** MDA-MB-231 cell line. **b** MCF-7 cell line. FXR was localized in the nucleus and around the nucleus in both cells lines. FXR expression increased after CDCA treatment (CDCA) compared to control cells (**c**). Scale bars = 100 μm

FXR expression was detected in MDA-MB-231 and MCF-7 cells by immunofluorescence, which is illustrated in Fig. 2-a and b, respectively. FXR was strongly expressed in the nucleus and lightly in the cytoplasm. CDCA treatment for 24 h (Fig. 2, right panels) caused a strong increase of nuclear FXR expression compared to control cells (Fig. 2, left panels). Some cells presented a strong immunoreactive pole near the nucleus after CDCA treatment.

### Bone protein expression in breast cancer cells

To establish the FXR implication in the osteomimetism of cells which could explain the osteotropism of breast cancer, immunofluorescences were performed (Supplemental Fig. 1, 2, 3, 4, 5, 6 and 7) on bone proteins after different treatments. Chenodeoxycholic acid (CDCA) is a primary bile acid and a strong agonist of FXR. Z-guggulsterone is a steroid acting as an antagonist of FXR [31]. LCA is a bile acid acting as a FXR competitive antagonist. This bile acid inhibits the FXR activation in presence of CDCA [31]. 4-hydroxytamoxifen (a partial antiestrogen) and fulvestrant (a pure antiestrogen) were used to inhibit ER in MCF-7 cells. CDCA was used at 2 10^− 5^ M, Z-guggulsterone at 4 10^− 5^ M and LCA at 8 10^− 5^ M. For MCF-7 cells, additional effectors were used, including E_2_ at 10^− 9^ M, 4-hydroxytamoxifen at 10^− 7^ M and fulvestrant at 10^− 7^ M.

**Fig. 3:**
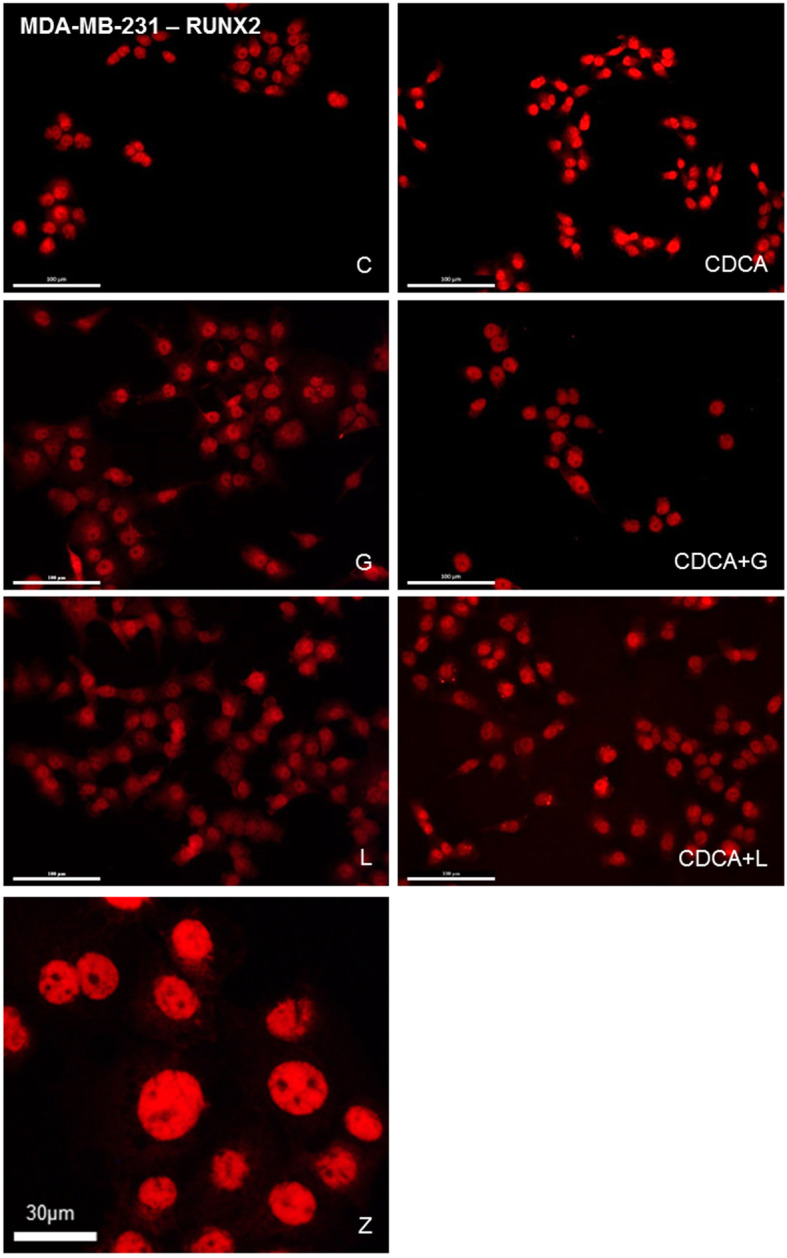
Treatments performed on MDA-MB-231 cell line: C = control, G = Z-guggulsterone, L = LCA, CDCA = chenodeoxycholic acid. Z: high magnification of a culture exposed to CDCA (Scale bar 30 μm). RUNX2 detection by immunofluorescence after different treatments during 24 h in MDA-MB-231. RUNX2 was evidenced in the nucleus. Z-guggulsterone (**g**) and LCA (**l**) caused no variation in RUNX2 expression compared to the control (**c**). CDCA treatment (CDCA) induced an increase of RUNX2 expression compared to the control (**c**). Z-guggulsterone or LCA in combined with CDCA (CDCA+G or CDCA+L) induced a decrease in RUNX2 expression compared to CDCA (CDCA). Scale bars = 100 μm. Immunofluorescence image panels of OPN, OC and BSP after equivalent treatments on MDA-MB-231 cell line were illustrated as supplementary Figures 1, 2 and 3. Immunofluorescence image panels of RUNX2, OPN, OC and BSP after equivalent treatments on MCF7 cell line were illustrated as supplementary Figures 4, 5, 6 and 7

Fig. 3 illustrated the effect of different treatments during 24 h on RUNX2 expression in MDA-MB-231. RUNX2 was located in the nucleus (Z). An activation of FXR by CDCA (CDCA) induced an increase of RUNX2 expression versus control cells (C) in MDA-MB-231. FXR inhibitory compounds such as z-guggulsterone (G) and lithocholic acid (L) had no impact on RUNX2 expression compared to the control (C). However, CDCA combined with z-guggulsterone (CDCA+G) or lithocholic acid (CDCA+L) caused a decrease of RUNX2 immunoreactivity compared to CDCA used alone.

**Fig. 4:**
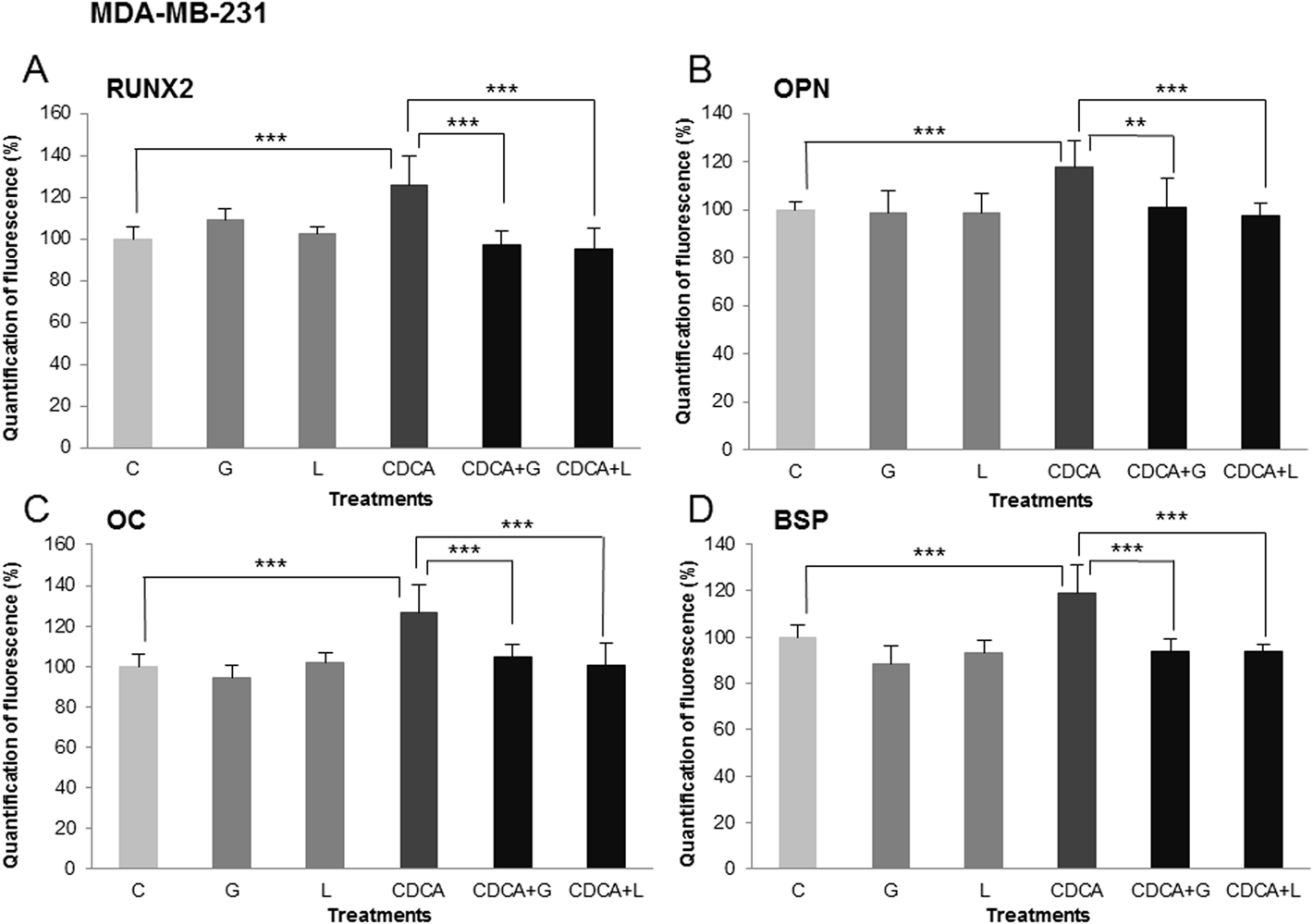
Treatments performed on MDA-MB-231 cell line: C = control, G = Z-guggulsterone, L = LCA, CDCA = chenodeoxycholic acid. **a** RUNX2, **b** OPN, **c** OC and **d** BSP respective expressions after different treatments. Values represent the mean percentage of mean fluorescence ± SEM of nine independent experiments. For all four markers, the percentage of fluorescence increased significantly after CDCA treatment compared to control values normalized at 100%. The inhibitor agents (LCA, Z-guggulsterone) in combination with CDCA decreased the percentage of fluorescence compared to CDCA used alone (*** *p* < 0.001, ** *p* < 0.01)

**Fig. 5:**
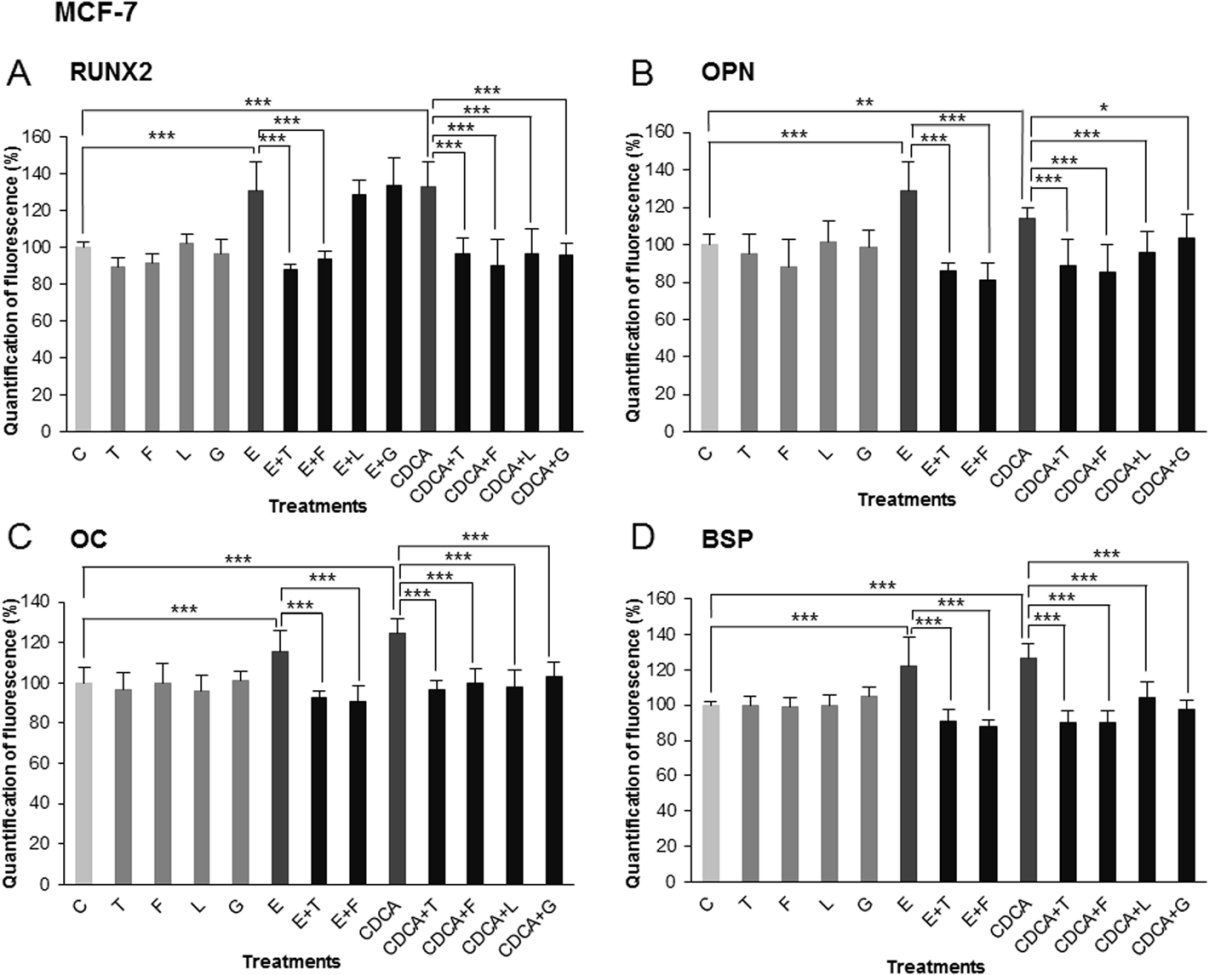
Treatments performed on MCF7 cell line: C = control, T = 4-hydroxitamoxifen, F = fulvestrant, L = LCA, E = estrogens, CDCA = chenodeoxycholic acid. **a** RUNX2, **b** OPN, **c** OC and **d** BSP respective expressions after different treatments. Values represent the mean percentage of mean fluorescence ± SEM of nine independent experiments. For all four markers, the percentage of fluorescence increased after CDCA or estrogens treatment compared to the control values (normalized at 100%). 4-Hydroxytamoxifen and fulvestrant caused an inhibition of the stimulating effect mediated by estrogens. 4-Hydroxytamoxifen, fulvestrant, LCA and Z-guggulsterone administered in combination with CDCA significantly decreased the percentage of fluorescence compared to CDCA used alone (*** *p* < 0.001, ** *p* < 0.01, * *p* < 0.05)

These effects were quantified by morphometry using image processing software. Measures of immunofluorescence intensity were done on nine fields of three independent cultures for all treatments and control and are illustrated in Figs. 4 and 5 (RUNX2, OPN, OC and BSP) for MDA-MB-231 and MCF-7, respectively. In MDA-MB-231 cells (Fig. 4-a, b, c and D), Z-guggulsterone (G) and LCA (L) caused no significant variation compared to the control. CDCA treatment (CDCA) for 24 h caused an increase of RUNX2 expression of 25% compared to the control (17% for OPN, 26% for OC, 19% for BSP). Z-guggulsterone and LCA significantly inhibited the effects of CDCA on bone protein expression (CDCA+G, CDCA+L). Indeed, in presence of both inhibitors, bone protein immunofluorescence levels were similar to control values without CDCA. For MCF-7 cells (Fig. 5-a, b, c and d), measures of immunofluorescence intensity of RUNX2, OPN, OC and BSP were performed and show the following: (i) a significant stimulation by E_2_ (E) (31% for RUNX2, 28% for OPN, 15% for OC, 21% for BSP) and CDCA (CDCA) (33% for RUNX2, 14% for OPN, 24% for OC, 26% for BSP); (ii) no effect of 4-hydroxytamoxifen (T), fulvestrant (F), Z-guggulsterone (G) and LCA (L) used alone; (iii) a total inhibition of E_2_/CDCA stimulation in presence of 4-hydroxytamoxifen (E + T, CDCA+T) and fulvestrant (E + F, CDCA+F) as well as LCA (CDCA+L) and Z-guggulsterone (CDCA+G). Stimulatory effects of estradiol were thus only inhibited by antiestrogens, whereas stimulatory effects of CDCA were inhibited both by FXR antagonists and by antiestrogens.

**Fig. 6:**
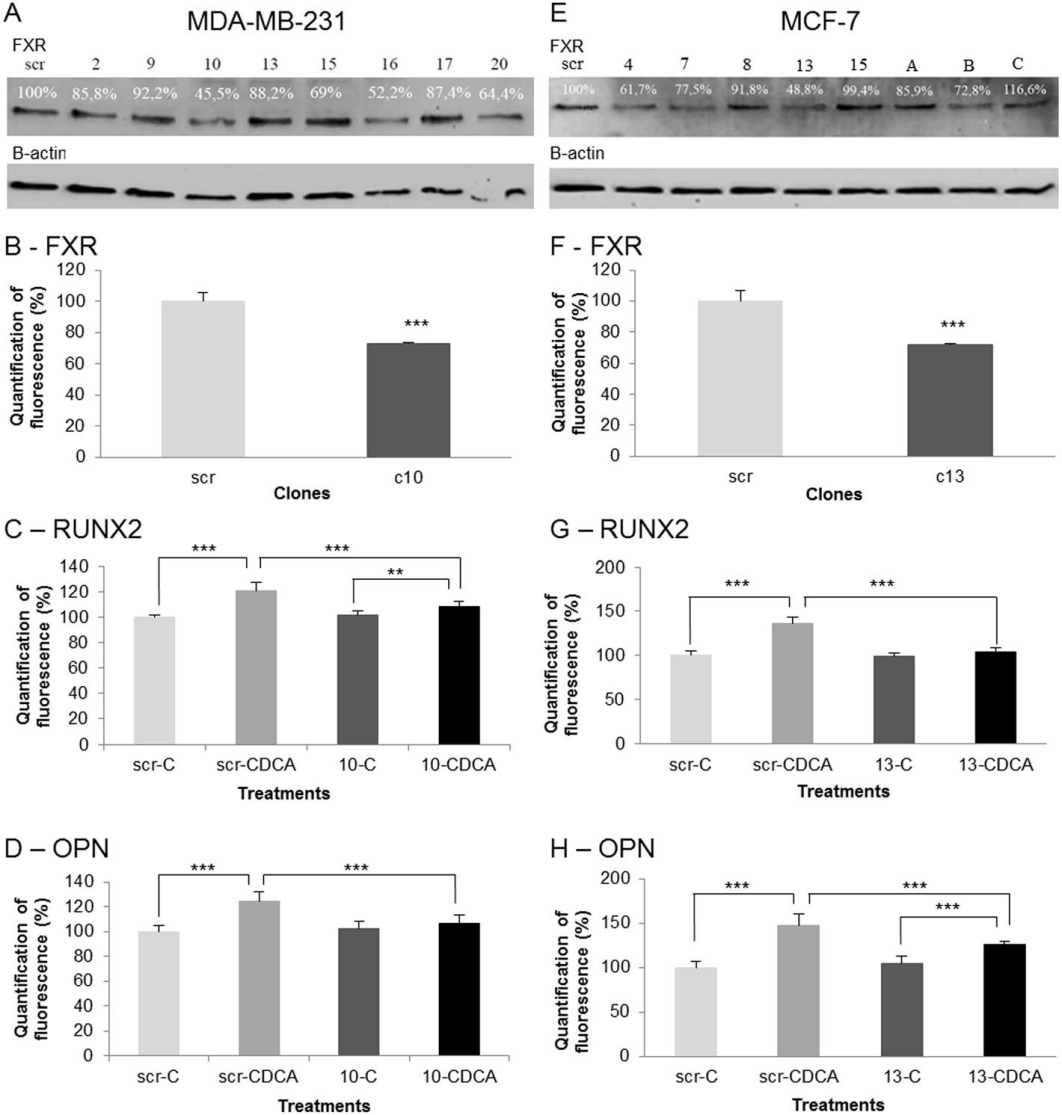
Effect of FXR knock down on the synthesis of bone proteins. Scr: scramble, 10 = clone 10, 13 = clone 13. **a** Western blotting of FXR in MDA-MB-231 cells after exposure to shRNA. Immuno bands are quantified and normalized with β-actin expression. Immunoreactive band intensities were quantified using the software ImageJ®. Full-length blots are presented in supplementary Figure 8**b** FXR expression and quantification in MDA-MB-231 cells after shRNA. **c** RUNX2 respective expressions after CDCA treatment in in MDA-MB-231. **d** OPN respective expressions after CDCA treatment in MDA-MB-231. **e** Western blotting of FXR in MCF-7 cells after shRNA. Values are quantified and normalized on β-actin expression. Immunoreactive band intensities were quantified using the software ImageJ®. Full-length blots are presented in supplementary Figure 9**f** FXR expression and quantification in MCF-7 cells after exposure to shRNA. **g** RUNX2 respective expressions after CDCA treatment in in MCF-7. **h** OPN respective expressions after CDCA treatment in MCF-7. Clone 10 and 13 showed the lowest expression in MDA-MB-231 and MCF-7 respectively. The decrease of FXR expression caused by shRNA significantly block the induction of RUNX2 and OPN expression reduction after CDCA treatment in clones compared to scrambles. (*** *p* < 0.001, ** *p* < 0.01)

The involvement of FXR in the expression of bone protein was further validated by using specific shRNA against FXR. The results are illustrated in Fig. 6. We selected clone 10 for MDA-MB-231 cells and clone 13 for MFC-7 cells. These clones showed the strongest inhibition of FXR in western blot and in immunofluorescence (Fig. 6-a, b, e and f). As expected, the FXR activation by CDCA caused an increase of RUNX2 and OPN in the negative control (scramble, scr) of MDA-MB-231 and MCF-7 lines. In shRNA clones, we showed a little increase or no increase of RUNX2 and OPN. No significant differences between scramble and the shRNA clone without CDCA treatment were observed. Conversely, a significant decrease of bone protein expression was observed under CDCA stimulation. Indeed, bone protein levels were significantly reduced in MDA-MB-231(clone10-CDCA) and MCF-7 (clone 13-CDCA) where the FXR was quenched by shRNA compared to controls (scr-CDCA) (Fig. 6-c, d, g and h). These data validate the FXR involvement in bone protein expression.

### Interaction between ER and FXR

**Fig. 7:**
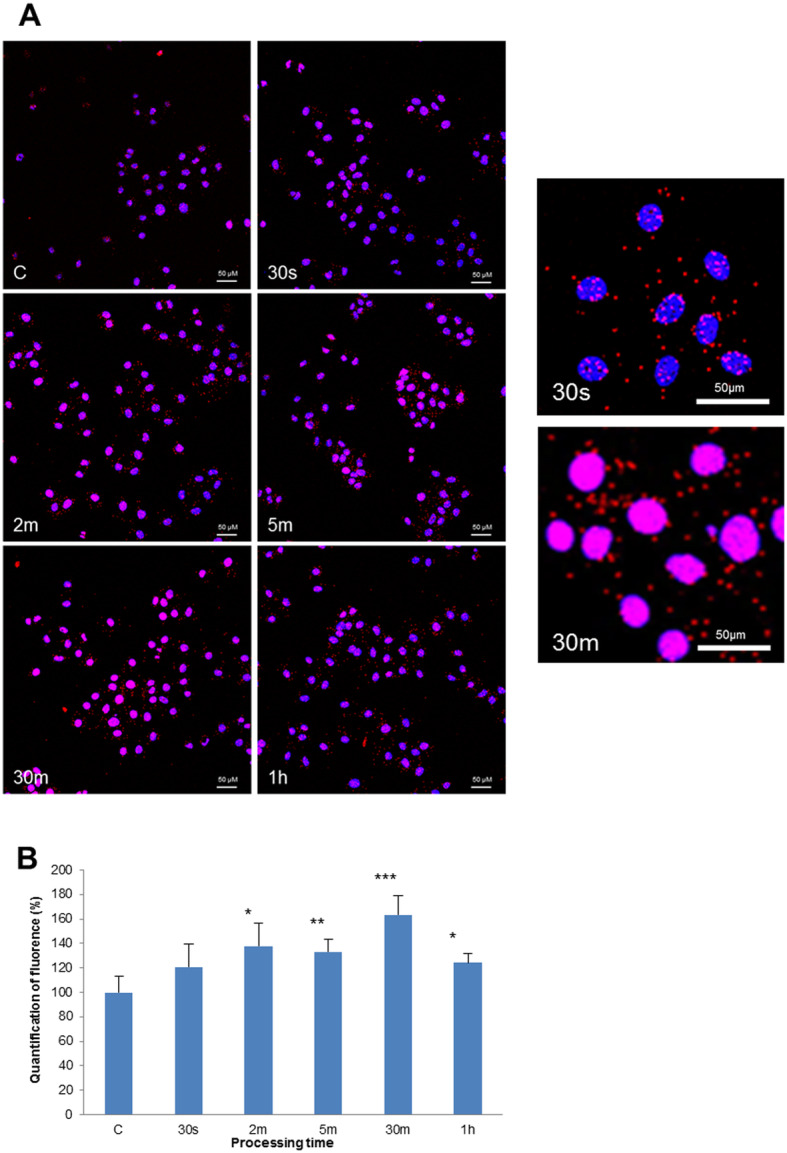
Molecular direct interaction between FXR and ER was evaluated by Proximity Ligation Assay (PLA) in the MCF7 cell line (**a**). The formation of heterodimers between both receptors (ER linked to FXR) was evaluated after exposure to CDCA (2 10^− 5^ M) at different time laps (from 30 s to 1 h). The heterodimers appeared as red spots and the nucleus was stained in blue by DAPI. In controls without CDCA stimulation (**c**), a low level of ER-FXR heterodimers was evidenced. After 2 min of CDCA (2 m), the number of heterodimers increased a lot in cells and started to translocate in the nucleus at 5 min (5 m). The maximum of heterodimers in the nucleus was observed at 30 min after CDCA treatment (30 m) and decreased after 1 h of CDCA (1 h). Scale bars = 50 μm. **b** ER-FXR dimers quantification. For all processing times, the percentage of dimers ER-FXR increased after a CDCA treatment compared to the control. The maximum was noticed at 30 m and decreased at 1 h of treatment. Values represent the mean percentage of mean fluorescence ± SEM of four independent experiments. (*** *p* < 0.001, ** *p* < 0.01, * *p* < 0.05). Control values are normalized at 100%

An interaction between FXR and ER was shown by using a Proximity Ligation Assay (PLA) in the MCF7 cells. The impact of CDCA (2 10^− 5^ M) during increasing exposure times (from 30 s to 1 h) is illustrated in Fig. 6. In control conditions (Fig. 7-a, left upper panel, c), a low level of ER-FXR heterodimers was evidenced. The sparse heterodimers were located both in the cytoplasm and nucleus of MCF-7 cells. After 2 min of CDCA treatment, the number of heterodimers markedly increased (Fig. 7-a, middle panels) and started to translocate in the nucleus at 5 min. The maximum amount of heterodimers in the nucleus was observed at 30 min after CDCA treatment and decreased after 1 h of CDCA (Fig. 7-a, lower panels). These observations were quantified by morphometry using image processing software (Fig. 7-b). Immunofluorescence intensity measures of the dimers ER-FXR were carried out on four independent cultures for all treatments and control and are illustrated in Fig. 7-b. The number of ER-FXR dimers started to increase at 30 s of CDCA treatment and was maximal at 30 min (+ 63%) of CDCA treatment. These data suggest the presence of a physical link between FXR and ER after exposure of MCF-7 cells to CDCA.

## Discussion

Some recent studies attest that the nuclear receptor FXR plays a role in the development and progression of cancer. However, the results of these studies are contradictory, some have evidenced an oncogenic function of FXR, while other works present FXR as a tumor suppressor [23, 32–35]. In breast cancer, some investigations indicate that FXR acts as oncogene able to induce an increase of malignant cell proliferation and to promote metastatic activity. Indeed, some studies show a correlation between high FXR expression and an increase in the cell proliferation rate evidenced by Ki-67 [16, 18]. In contrast, other authors have highlighted the opposite results and have attested that FXR caused a significant decrease in motility, invasion capacity and the tumor cell proliferation rate [36, 37]. Moreover, these studies suggested that FXR is also able to induce the apoptosis of tumor cells. In light of these contradictory results, it seems important to investigate the functions of FXR and the consequences of its activation in different estrogen or non-estrogen dependent breast cancer cell lines.

We targeted FXR expression in breast tumor samples by immunohistochemistry (IHC). Our clinical study showed primary breast cancer with bone metastases had a median high score at 6 for FXR compared to visceral metastases (score = 4.5). Moreover, this difference is even more pronounced in aggressive tumors (histological grade 3) (score = 7 versus 2, respectively). Clinical data revealed an intriguing relationship between FXR expression in primary breast cancer and the propensity of these neoplastic cells to develop osteotropism and bone metastasis in the first place. If confirmed on large series, these results suggest that high FXR expression seems to predict bone metastases in aggressive tumors (positive predictive value of 91%) and that such a receptor could be used as a new predictive marker to select patients who should benefit from inhibitors of bone resorption, such as zoledronic acid [38].

Our experiment by immunofluorescence revealed that FXR was expressed both in MDA-MB-231 and in MCF-7 cell lines. This is not surprising because many studies attested to the presence of FXR in breast cancer, as well as in healthy breast tissue where FXR was detected both in alveolar and ductal epithelial cells [16, 18]. The receptor was located in the nucleus and lightly in the cytoplasm of cells. This nucleocytoplasmic distribution was similar in MDA-MB-231 and MCF-7 cells, and was in agreement with previous immunocytochemical studies [16, 18]. As reported in the literature, most nuclear receptor location is restricted to the nucleus, however some nuclear receptors are located in the cytoplasm, like androgen and glucocorticoid receptors [39, 40]. Many FXR agonists are used in in vitro experiments and some molecules are used in clinical hepatobiliary studies [41]. Bile acids are the physiological ligands of FXR, and CDCA is the most potent agonist [42]. Our experiment showed an increase of FXR expression in both cell lines after stimulation by CDCA, suggesting an upregulation of the nuclear receptor. Most hormonal receptors have a downregulation after activation by the ligand, like estrogen and insulin receptors. However, the upregulation of FXR is not surprising because this effect is normally observed in the liver [43]. Furthermore, this result joins the works showing an upregulation of FXR after stimulation by CDCA or farnesol, another agonist of FXR [18, 44].

Indeed, by binding to FXR, CDCA could activate an FXR/ER heterodimer and promote significant cell proliferation through its interaction with the estrogen response element (ERE). The direct molecular interaction between FXR and ER that we demonstrated in the MCF-7 line, using the PLA method, seems to corroborate this hypothesis. This FXR/ER interaction was also highlighted in previous publications of our group by a method of co-immunoprecipitation [18, 44].

FXR antagonists are numerous and are both natural and synthetic [31]. Guggulsterone is a vegetal sterol derived from the resin of an Asian tree *Commiphora wightii* and has been consumed in traditional medicine for centuries to treat obesity, liver disorders and a large panel of tumors [45]. This molecule is often used in preclinical in vitro and in vivo studies and showed a decrease of FXR activity and the tumor size [46]. The second FXR inhibitor that we used was LCA. Its competitive inhibition capacity has been documented in studies dedicated to the FXR activation pathway. Indeed, the affinity for FXR depends on its hydrophobicity and LCA is more hydrophobic than CDCA [20, 47].

Bone is frequently invaded by cancer cells, in particular those coming from breast and prostate cancers. Synthesis and expression of bone matrix proteins by breast cancer cells mimic bone tissue. This osteomimetism could explain the tropism for these cancer cells to metastasize in bone. In this study, we have examined the involvement of FXR in the osteomimetism of breast cancer cells. We have shown that FXR activation by CDCA induced an increase of RUNX2 and other bone proteins. Conversely, Z-guggusterone and LCA used alone did not change the expression of bone proteins. However, in combination with CDCA, these two effectors inhibited the positive stimulation of CDCA. Using shRNA against FXR, we validated our results showing that FXR stimulated the osteomimetism of breast cancer cells. RUNX2 is a transcription factor that transactivates the synthesis of OPN, OC and BSP [27]. Therefore, it is not unusual to find changes in bone protein expressions after activation or inhibition of RUNX2. Similar regulation exerted by RUNX2 has been reported in the bone forming cell osteoblasts. Furthermore, RUNX2 is also involved in the induction of the proliferation and differentiation of osteoblast progenitor cells [48]. Cho and collaborators [49] recently demonstrated that FXR is expressed both in osteoblasts and bone marrow stem cells. Moreover, FXR depletion caused a decrease of osteogenesis in vivo. In the same study, the authors showed that CDCA treatment increased the expression of some bone markers, like RUNX2 [49]. In another study, Id Boufker and collaborators [50] evidenced a high level of mRNA coding for FXR in bone marrow stroma cells and SaOS2 (pre-osteoblastic cells). This research demonstrated an increase of calcium associated with the extracellular matrix after CDCA treatment. FXR activation also induced an increase of bone markers (OPN, OC and BSP) and this stimulation was inhibited by guggulsterone. Finally, shRNA against FXR failed to stimulate bone marker expressions after CDCA treatment [50]. On the other hand, in the present study, the inhibition of 50% FXR expression in shRNA selected clones induces a significant but not spectacular decrease of bone protein expression after simulation by CDCA. This moderate impact could be explained by the fact that the targeted bone proteins (OPN, OC and BSP) depend on the expression of RUNX2 but FXR is not the only protein causing the activation of RUNX2. Many other nuclear receptors activate RUNX2, such as the vitamin D receptor and the estrogen receptor. We could suspect that these other signal pathways have offset the decrease of FXR expression and therefore the transactivation of RUNX2. Recent studies (see [51] for review) have also demonstrated that RUNX2 can also be activated by several kinases such as ERK, PIM-1 and PI3K. Furthermore, RUNX2 gene contains transcription factor AP-1 and NF-1 binding site [52].

Both breast cancer cell lines (MDA-MB-231 and MCF-7) that were tested in the present study showed a very similar pattern to bone cells when simulated by FXR agonists or inhibited by antagonists. Indeed, these malignant mammary cell lines overexpressed bone proteins after FXR activation. That could explain the propensity of breast cancer cells to metastasize in bone. However, it is not surprising that estrogens also stimulated RUNX2 and the other bone proteins. Indeed, estrogens are essential in bone homeostasis and osteoblast differentiation, and they inhibit the bone resorption [27, 53]. Furthermore, ER interacts with RUNX2 promotor and stimulates gene transcription [27].

Finally, recent studies have attested that RUNX2 plays a key role in the interaction between breast cancer cells and the bone microenvironment. The authors evidenced that BSP was associated with micro calcification observed in some primary breast tumors and promoted bone metastasizes. They also pointed to the involvement of BSP in the process of invasion and progression of tumor cells [54–56].

**Fig. 8:**
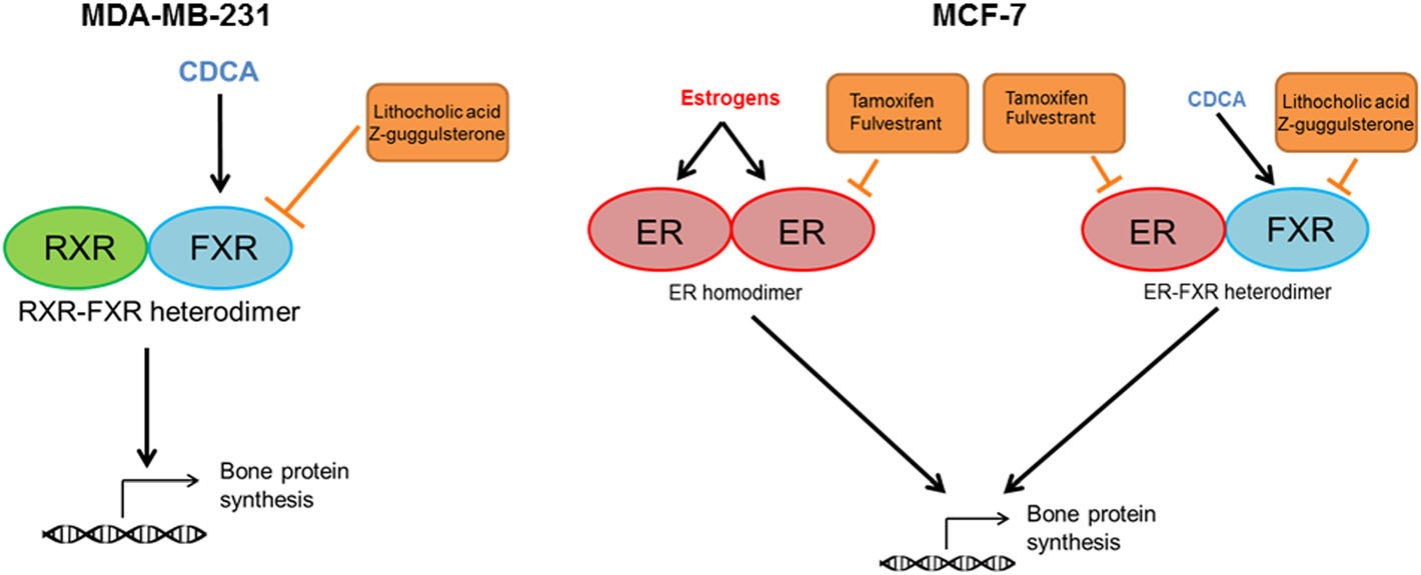
Regulation of bone protein synthesis in breast cancer cell lines. In MDA-MB-231 cells, CDCA binds FXR. FXR dimerizes with another nuclear receptor and activates the bone protein synthesis. In MCF-7 cells, estrogens bind ER and dimerizes with another estrogen receptor. When CDCA is used and binds FXR, FXR dimerizes with ER. The homodimer ER-ER and the heterodimer FXR-ER both activate the bone protein synthesis

Altogether, clinical data associated FXR expression in breast cancers and the development of bone metastases. Experimental data highly validate clinical data and support, on the one hand, a crosstalk between FXR and RUNX2 expression/activation in MDA-MB-231 cells and, on the other hand, a crosstalk between FXR, ER and RUNX2 expression/activation in MCF-7 cells. These results suggest an implication of FXR (in MDA-MB-231 cells) and a synergetic involvement of FXR and ER (in MCF-7 cells) in the osteomimetism of breast cancer cells. Figure 8 summarizes the data and hypothesis. In MDA-MB-231, FXR activation by CDCA induces its dimerization, probably with the classical RXR nuclear receptor. The heterodimer activates the transcription of RUNX2 leading to the transcription of genes coding for bone matrix proteins. LCA and Z-guggulsterone inhibit FXR activation and validate the role of FXR in this mechanism. In MCF-7 cells, the transactivation of RUNX2 can be induced by two pathways. When estrogens are used, ER is activated and dimerizes with another ER. This homodimer induces the synthesis of RUNX2. Anti-estrogens prevent and prove this effect. In the case of CDCA treatment, FXR is activated and dimerizes with ER as demonstrated by the PLA analysis. The heterodimer FXR-ER transactivates RUNX2 expression. Tamoxifen and fulvestrant inhibit this action, targeting ER, while Z-guggulsterone and LCA prevent the activation of FXR, all inhibitors validating these mechanisms.

## Conclusions

Results of clinical study point to a relationship between the expression of FXR in breast primary tumor and the propensity of these malignant cells to develop bone metastases. In vitro study performed on breast cancer cell lines (MDA-MB-231 and MCF-7) demonstrates that these tumoral cells showed a very similar pattern to bone cells when simulated by FXR agonists or inhibited by antagonists. Indeed, these malignant mammary cell lines overexpressed bone proteins after FXR activation. This osteomimetism could explain the propensity of breast cancer cells to metastasize in bone. Therefore, FXR should be viewed as a new marker to predict bone metastases and as a new target for breast cancer therapies.

## Supplementary information

**Additional file 1: Supplementary Figure 1.** Osteopontin (OPN) expression after different treatments during 48 h in MDA-MB-231. OPN immunostaining was evidenced in the cytoplasm. Z-guggulsterone (G) and LCA (L) caused no variation in OPN expression compared to the control (C). CDCA treatment (CDCA) induced an increase of OPN expression compared to the control (C). Z-guggulsterone or LCA in combination with CDCA (CDCA+G or CDCA+L) caused a decrease of OPN expression versus CDCA (CDCA) alone. Scale bars = 100 μm.

**Additional file 2: Supplementary Figure 2.** OC expression after different treatments during 48 h in MDA-M-231. OC was evidenced by immunofluorescence and is expressed in the cytoplasm. Z-guggulsterone (G) and LCA (L) caused no variation in OC expression compared to the control (C). CDCA treatment (CDCA) induced an increase of OC expression compared to the control (C). Z-guggulsterone or LCA in combined with CDCA (CDCA+G or CDCA+L) caused a decrease in OC expression compared to CDCA (CDCA). Scale bars = 100 μm.

**Additional file 3: Supplementary Figure 3.** BSP expression after different treatments during 48 h in MDA-M-231. BSP was evidenced by immunofluorescence. BSP is expressed in the cytoplasm and appeared as a pole in proximity of the nucleus. Z-guggulsterone (G) and LCA (L) caused no variation in BSP expression compared to the control (C). CDCA treatment (CDCA) induced an increase of BSP expression compared to the control (C). Z-guggulsterone or LCA in combined with CDCA (CDCA+G or CDCA+L) caused a decrease in BSP expression compared to CDCA (CDCA). Scale bars = 100 μm.

**Additional file 4: Supplementary Figure 4.** RUNX2 expression after different treatments during 24 h in MCF-7. RUNX2 was evidenced by immunofluorescence and was localized in the nucleus. Estrogens (E) and CDCA (CDCA) induced an increase of RUNX2 expression compared to the control (C). 4-hydroxytamoxifen (T), fulvestrant (F), LCA (L) and Z-guggulsterone (G) caused no variation of RUNX2 expression compared to the control (C). 4-hydroxytamoxifen and fulvestrant co-administered with estrogens or with CDCA (E + T, E + F, CDCA+T, CDCA+F) caused a decrease of RUNX2 expression versus estrogens (E) or CDCA (CDCA) used alone. LCA and Z-guggulsterone used in combination with estrogens (E + L, E + G) induced no variation of RUNX2 expression versus an exposure to estrogens (E) alone. LCA and Z-guggulsterone co-administered with CDCA (CDCA+L, CDCA+G) elicited a decrease of RUNX2 expression compared to CDCA (CDCA) used alone. Scale bars = 100 μm.

**Additional file 5: Supplementary Figure 5.** Osteopontin (OPN) evidenced by immunofluorescence after different treatments during 48 h in MCF-7. OPN-immunostaining was localized in the cytoplasm. Estrogens (E) and CDCA (CDCA) induced an increase of OPN expression compared to the control (C). 4-hydroxytamoxifen (T), fulvestrant (F), LCA (L) and Z-guggulsterone (G) caused no variation in OPN expression compared to the control (C). 4-hydroxytamoxifen, fulvestrant, LCA and Z-guggulsterone co-administered with CDCA (CDCA+T, CDCA+F, CDCA+L, CDCA+G) caused a decrease of OPN expression versus CDCA used alone. 4-hydroxytamoxifen or fulvestrant used in combination with estrogens (E + T, E + F) induced a decrease of OPN expression versus an exposure to estrogens (E) alone. Scale bars = 100 μm.

**Additional file 6: Supplementary Figure 6.** OC expression after different treatments during 48 h in MCF-7. OC was evidenced by immunofluorescence and is expressed in the cytoplasm. Estrogens (E) and CDCA (CDCA) induced an increase of OC expression compared to the control (C). 4-hydroxytamoxifen (T), fulvestrant (F), LCA (L) and Z-guggulsterone (G) caused no variation in OC expression compared to the control (C). 4-hydroxytamoxifen and fulvestrant in combined with estrogens or CDCA (E + T, E + F, CDCA+T, CDCA+F) caused a decrease of OC expression compared to estrogens (E) or CDCA (CDCA). LCA and Z-guggulsterone in combined with CDCA (CDCA+L, CDCA+G) caused a decrease in OC expression compared to CDCA (CDCA). Scale bars = 100 μm.

**Additional file 7: Supplementary Figure 7.** BSP expression after different treatments during 48 h in MCF-7. BSP was evidenced by immunofluorescence and is expressed in the cytoplasm. Estrogens (E) and CDCA (CDCA) induced an increase of BSP expression compared to the control (C). 4-hydroxytamoxifen (T), fulvestrant (F), LCA (L) and Z-guggulsterone (G) caused no variation in BSP expression compared to the control (C). 4-hydroxytamoxifen and fulvestrant in combined with estrogens or CDCA (E + T, E + F, CDCA+T, CDCA+F) caused a decrease of BSP expression compared to estrogens (E) or CDCA (CDCA). LCA and Z-guggulsterone in combined with CDCA (CDCA+L, CDCA+G) caused a decrease in BSP expression compared to CDCA (CDCA). Scale bars = 100 μm.

**Additional file 8: Supplementary Figure 8.** Full-length Western Blotting with the cropped area corresponding to the image illustrated in Fig. 6a of the main text. Effect of FXR knock down on the synthesis of bone proteins. Scr: scramble, tested clones (2,9,10,13,15,16,17,20). **A**: Western blotting of FXR in MDA-MB-231 cells after exposure to shRNA. Immuno bands are quantified and normalized with β-actin expression (illustrated in full blot **B**). Immunoreactive band intensities were quantified using the software ImageJ®.

**Additional file 9: Supplementary Figure 9.** Full-length Western Blotting with the cropped area corresponding to the image illustrated in Fig. 6e of the main text. Effect of FXR knock down on the synthesis of bone proteins. Scr: scramble, tested clones (4, 7, 8, 13, 15, A, B, C). A: Western blotting of FXR in MCF-7 cells after exposure to shRNA. Immuno bands are quantified and normalized with β-actin expression (illustrated in full blot B). Immunoreactive band intensities were quantified using the software ImageJ®.

**Additional file 10.**

## Data Availability

Most of the data used to support the findings of this study are included within the article. Some pictures illustrating RUNX2, OPN, OC and BSP immunofluorescence are presented in the annexes as additional figures.

## Abbreviations

ADN: Acide désoxyribonucléique
BSP: Bone sialoprotein
CDCA: Chenodeoxycholic acid
CYP7A1: Cytochrome P450 7A1
DMEM: Dulbecco’s modified Eagle’s medium
EDTA: Ethylenediaminetetraacetic acid
ER: Estrogen receptor
ERE: Estrogen response element
F: Fulvestrant
FBS: Fetal bovine serum
FXR: Farnesoid X receptor
G: Z-guggulsterone
IHC: Immunohistochemistry
LCA: Lithocholic acid
MAPK: Mitogen-activated protein kinase
NSCLC: Non-small-cell lung carcinoma
OC: Ostéocalcine
OPN: Ostéopontine
PI3KCA: Phosphatidylinositol 3-kinase
PLA: Proximity ligation assay
RANK: Receptor activator of nuclear factor kappa-B
RANKL: Receptor activator of nuclear factor kappa-B ligand
RIPA: RadioImmunoPrecipitation Assay
RUNX2: Runt-related transcription factor 2
RXR: Retinoid X receptor
shRNA: Short Hairpin RNA
Src: Proto-oncogene tyrosine-protein kinase Src
T: 4-hydroxytamoxifen
TMA: Tissue microarrays
