# Supplementary material for: Farnesoid X receptor as marker of osteotropism of breast cancers through its role in the osteomimetism of tumor cells
Source: BMC Cancer. 2020 Jul 10;20:640. doi: 10.1186/s12885-020-07106-7 (PMC7350202; doi:10.1186/s12885-020-07106-7)
Supplement: Supplementary file 1 — Additional file 1: Supplementary Figure 1. Osteopontin (OPN) expression after different treatments during 48 h in MDA-MB-231. OPN immunostaining was evidenced in the cytoplasm. Z-guggulsterone (G) and LCA (L) caused no variation in OPN expression compared to the control (C). CDCA treatment (CDCA) induced an increase of OPN expression compared to the control (C). Z-guggulsterone or LCA in combination with CDCA (CDCA+G or CDCA+L) caused a decrease of OPN expression versus CDCA (CDCA) alone. Scale bars = 100 μm. [file 12885_2020_7106_MOESM1_ESM.pdf]

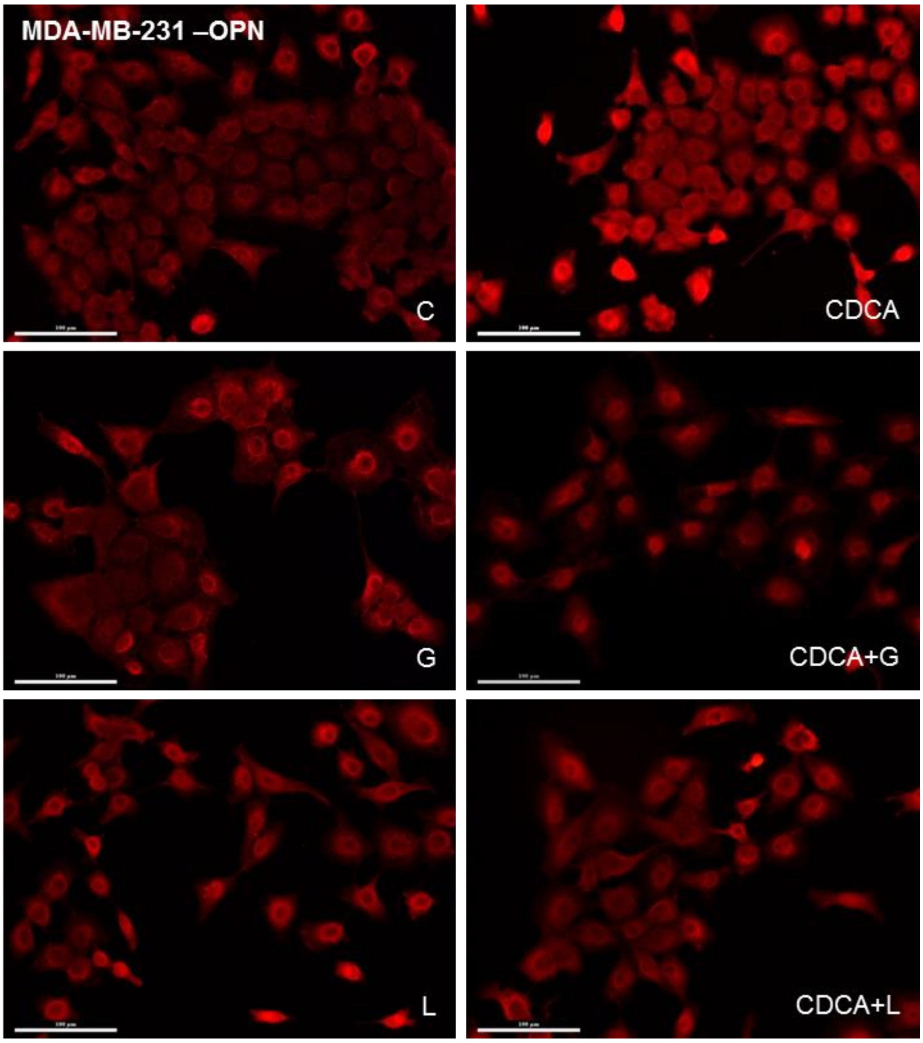

Supplementary Figure 1: Osteopontin (OPN) expression after different treatments during 48h in MDA-MB-231. OPN immunostaining was evidenced in the cytoplasm. Z-guggulsterone (G) and LCA (L) caused no variation in OPN expression compared to the control (C). CDCA treatment (CDCA) induced an increase of OPN expression compared to the control (C). Z-guggulsterone or LCA in combination with CDCA (CDCA+G or CDCA+L) caused a decrease of OPN expression versus CDCA (CDCA) alone. Scale bars = 100 µm.
