# Supplementary material for: Farnesoid X receptor as marker of osteotropism of breast cancers through its role in the osteomimetism of tumor cells
Source: BMC Cancer. 2020 Jul 10;20:640. doi: 10.1186/s12885-020-07106-7 (PMC7350202; doi:10.1186/s12885-020-07106-7)
Supplement: Supplementary file 2 — Additional file 2: Supplementary Figure 2. OC expression after different treatments during 48 h in MDA-M-231. OC was evidenced by immunofluorescence and is expressed in the cytoplasm. Z-guggulsterone (G) and LCA (L) caused no variation in OC expression compared to the control (C). CDCA treatment (CDCA) induced an increase of OC expression compared to the control (C). Z-guggulsterone or LCA in combined with CDCA (CDCA+G or CDCA+L) caused a decrease in OC expression compared to CDCA (CDCA). Scale bars = 100 μm. [file 12885_2020_7106_MOESM2_ESM.pdf]

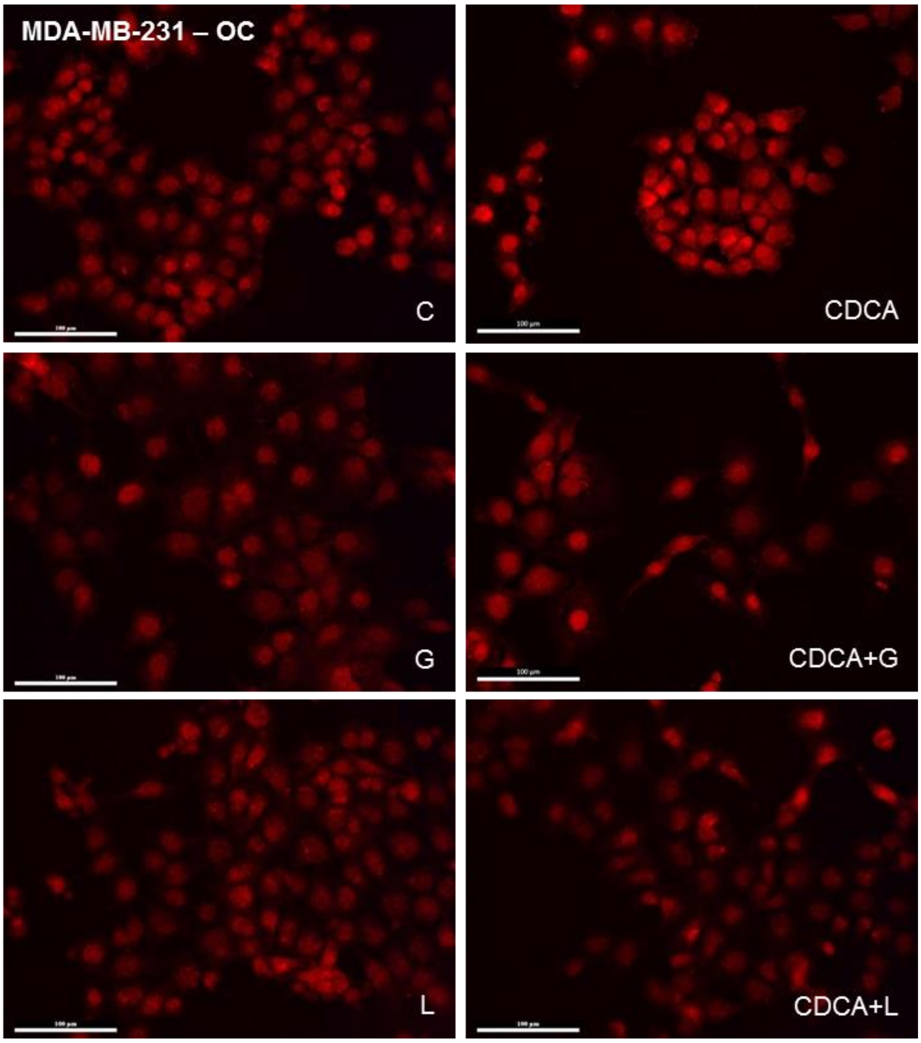

Supplementary Figure 2: OC expression after different treatments during 48h in MDA-M-231. OC was evidenced by immunofluorescence and is expressed in the cytoplasm. Z-guggulsterone (G) and LCA (L) caused no variation in OC expression compared to the control (C). CDCA treatment (CDCA) induced an increase of OC expression compared to the control (C). Z-guggulsterone or LCA in combined with CDCA (CDCA+G or CDCA+L) caused a decrease in OC expression compared to CDCA (CDCA). Scale bars = 100 µm.
