# Supplementary material for: Farnesoid X receptor as marker of osteotropism of breast cancers through its role in the osteomimetism of tumor cells
Source: BMC Cancer. 2020 Jul 10;20:640. doi: 10.1186/s12885-020-07106-7 (PMC7350202; doi:10.1186/s12885-020-07106-7)
Supplement: Supplementary file 3 — Additional file 3: Supplementary Figure 3. BSP expression after different treatments during 48 h in MDA-M-231. BSP was evidenced by immunofluorescence. BSP is expressed in the cytoplasm and appeared as a pole in proximity of the nucleus. Z-guggulsterone (G) and LCA (L) caused no variation in BSP expression compared to the control (C). CDCA treatment (CDCA) induced an increase of BSP expression compared to the control (C). Z-guggulsterone or LCA in combined with CDCA (CDCA+G or CDCA+L) caused a decrease in BSP expression compared to CDCA (CDCA). Scale bars = 100 μm. [file 12885_2020_7106_MOESM3_ESM.pdf]

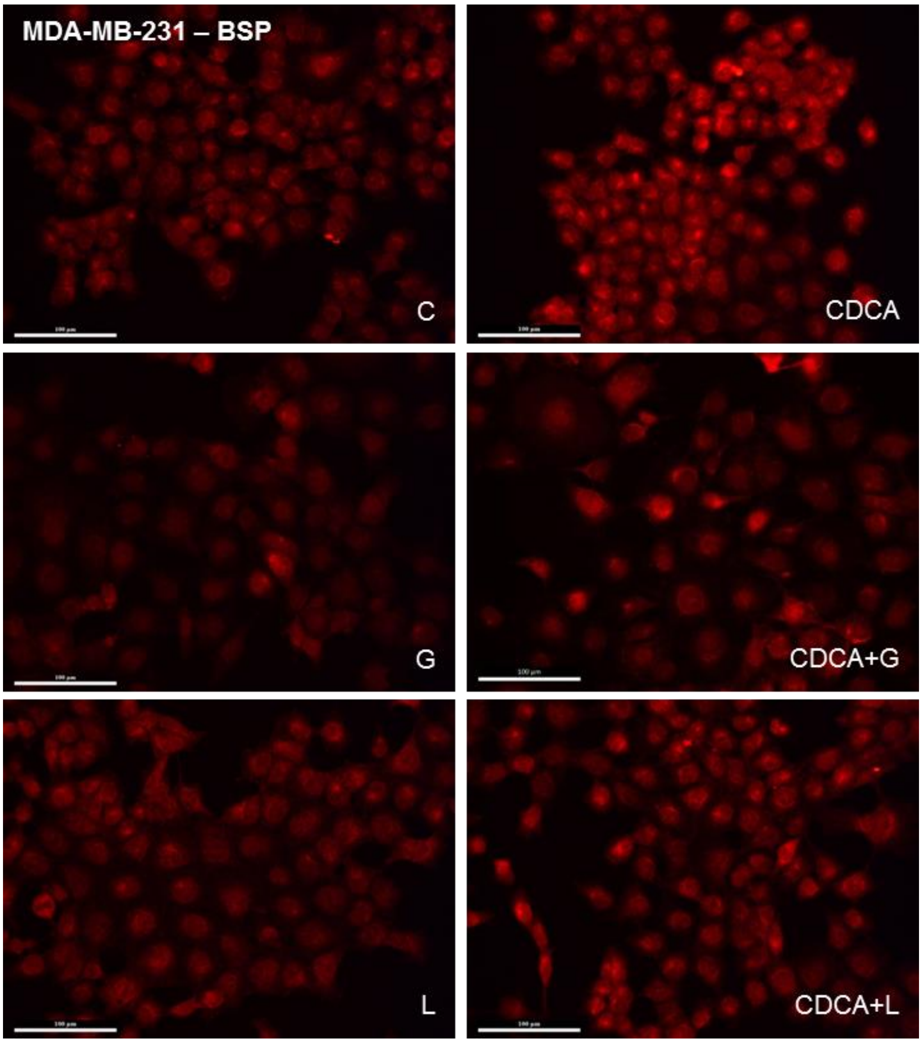

Supplementary Figure 3: BSP expression after different treatments during 48h in MDA-M-231. BSP was evidenced by immunofluorescence. BSP is expressed in the cytoplasm and appeared as a pole in proximity of the nucleus. Z-guggulsterone (G) and LCA (L) caused no variation in BSP expression compared to the control (C). CDCA treatment (CDCA) induced an increase of BSP expression compared to the control (C). Z-guggulsterone or LCA in combined with CDCA (CDCA+G or CDCA+L) caused a decrease in BSP expression compared to CDCA (CDCA). Scale bars = 100 µm.
