# Supplementary material for: Farnesoid X receptor as marker of osteotropism of breast cancers through its role in the osteomimetism of tumor cells
Source: BMC Cancer. 2020 Jul 10;20:640. doi: 10.1186/s12885-020-07106-7 (PMC7350202; doi:10.1186/s12885-020-07106-7)
Supplement: Supplementary file 4 — Additional file 4: Supplementary Figure 4. RUNX2 expression after different treatments during 24 h in MCF-7. RUNX2 was evidenced by immunofluorescence and was localized in the nucleus. Estrogens (E) and CDCA (CDCA) induced an increase of RUNX2 expression compared to the control (C). 4-hydroxytamoxifen (T), fulvestrant (F), LCA (L) and Z-guggulsterone (G) caused no variation of RUNX2 expression compared to the control (C). 4-hydroxytamoxifen and fulvestrant co-administered with estrogens or with CDCA (E + T, E + F, CDCA+T, CDCA+F) caused a decrease of RUNX2 expression versus estrogens (E) or CDCA (CDCA) used alone. LCA and Z-guggulsterone used in combination with estrogens (E + L, E + G) induced no variation of RUNX2 expression versus an exposure to estrogens (E) alone. LCA and Z-guggulsterone co-administered with CDCA (CDCA+L, CDCA+G) elicited a decrease of RUNX2 expression compared to CDCA (CDCA) used alone. Scale bars = 100 μm. [file 12885_2020_7106_MOESM4_ESM.pdf]

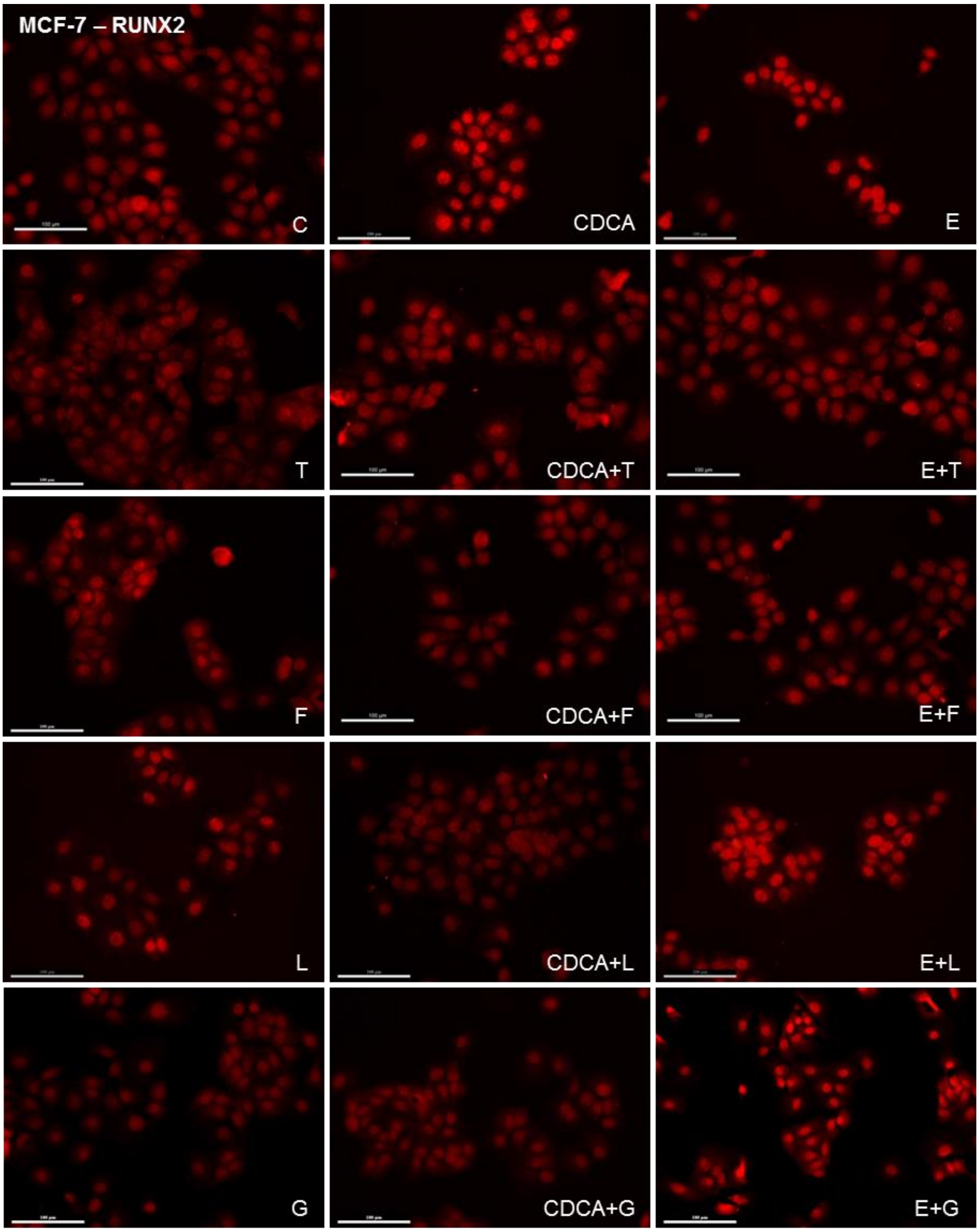

Supplementary Figure 4: RUNX2 expression after different treatments during 24h in MCF-7. RUNX2 was evidenced by immunofluorescence and was localized in the nucleus. Estrogens (E) and CDCA (CDCA) induced an increase of RUNX2 expression compared to the control (C). 4-hydroxytamoxifen (T), fulvestrant (F), LCA (L) and Z-guggulsterone (G) caused no variation of RUNX2 expression compared to the control (C). 4-hydroxytamoxifen and fulvestrant co-administered with estrogens or with CDCA (E+T, E+F, CDCA+T, CDCA+F) caused a decrease of RUNX2 expression versus estrogens (E) or CDCA (CDCA) used alone. LCA and Z-guggulsterone used in combination with estrogens (E+L, E+G) induced no variation of RUNX2 expression versus an exposure to estrogens (E) alone. LCA and Z-guggulsterone co-administered with CDCA (CDCA+L, CDCA+G) elicited a decrease of RUNX2 expression compared to CDCA (CDCA) used alone. Scale bars = 100 µm.
