# Supplementary material for: Farnesoid X receptor as marker of osteotropism of breast cancers through its role in the osteomimetism of tumor cells
Source: BMC Cancer. 2020 Jul 10;20:640. doi: 10.1186/s12885-020-07106-7 (PMC7350202; doi:10.1186/s12885-020-07106-7)
Supplement: Supplementary file 5 — Additional file 5: Supplementary Figure 5. Osteopontin (OPN) evidenced by immunofluorescence after different treatments during 48 h in MCF-7. OPN-immunostaining was localized in the cytoplasm. Estrogens (E) and CDCA (CDCA) induced an increase of OPN expression compared to the control (C). 4-hydroxytamoxifen (T), fulvestrant (F), LCA (L) and Z-guggulsterone (G) caused no variation in OPN expression compared to the control (C). 4-hydroxytamoxifen, fulvestrant, LCA and Z-guggulsterone co-administered with CDCA (CDCA+T, CDCA+F, CDCA+L, CDCA+G) caused a decrease of OPN expression versus CDCA used alone. 4-hydroxytamoxifen or fulvestrant used in combination with estrogens (E + T, E + F) induced a decrease of OPN expression versus an exposure to estrogens (E) alone. Scale bars = 100 μm. [file 12885_2020_7106_MOESM5_ESM.pdf]

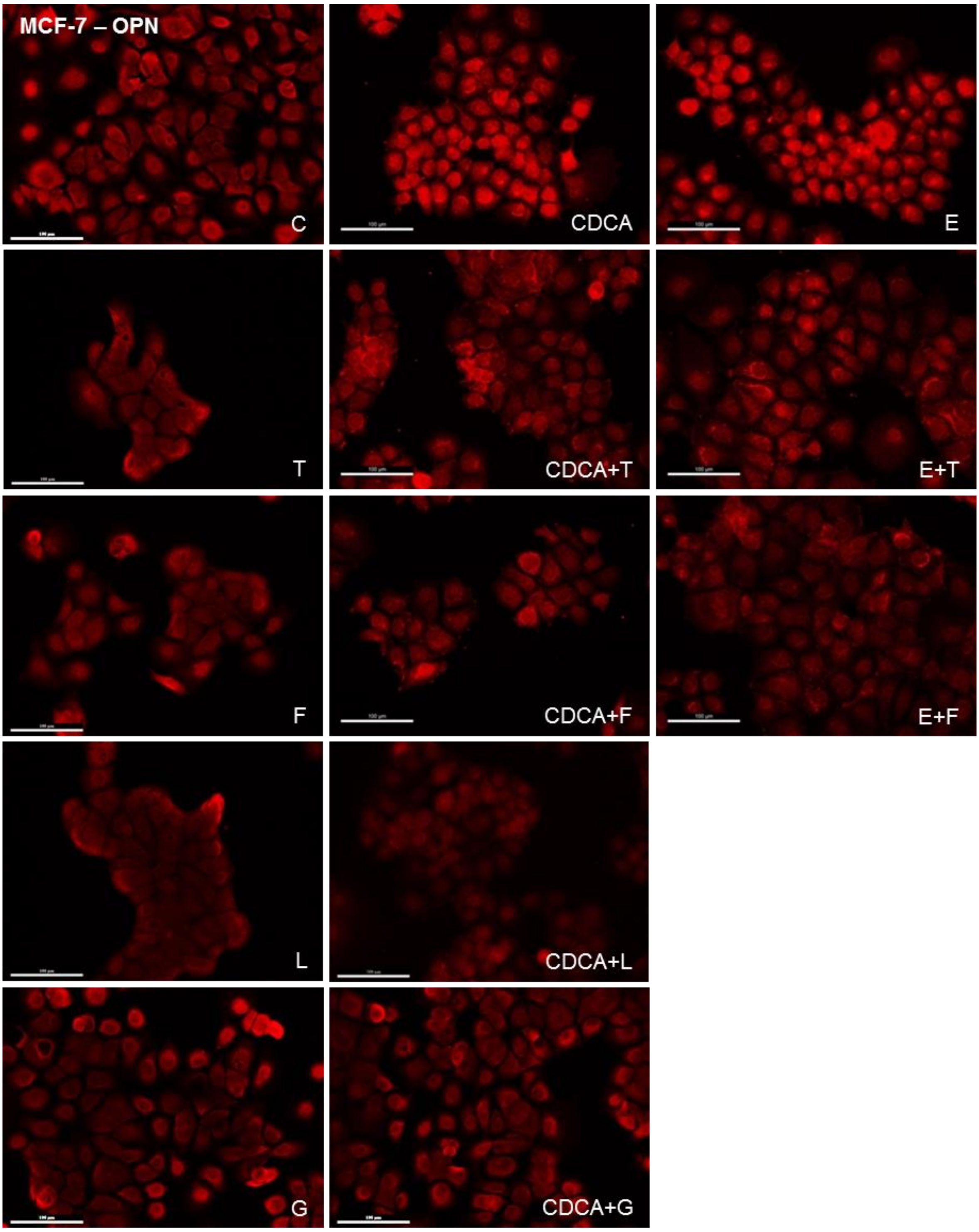

Supplementary Figure 5: Osteopontin (OPN) evidenced by immunofluorescence after different treatments during 48h in MCF-7. OPN-immunostaining was localized in the cytoplasm. Estrogens (E) and CDCA (CDCA) induced an increase of OPN expression compared to the control (C). 4-hydroxytamoxifen (T), fulvestrant (F), LCA (L) and Z-guggulsterone (G) caused no variation in OPN expression compared to the control (C). 4-hydroxytamoxifen, fulvestrant, LCA and Z-guggulsterone co-administered with CDCA (CDCA+T, CDCA+F, CDCA+L, CDCA+G) caused a decrease of OPN expression versus CDCA used alone. 4-hydroxytamoxifen or fulvestrant used in combination with estrogens (E+T, E+F) induced a decrease of OPN expression versus an exposure to estrogens (E) alone. Scale bars = 100 µm.
