# Supplementary material for: Farnesoid X receptor as marker of osteotropism of breast cancers through its role in the osteomimetism of tumor cells
Source: BMC Cancer. 2020 Jul 10;20:640. doi: 10.1186/s12885-020-07106-7 (PMC7350202; doi:10.1186/s12885-020-07106-7)
Supplement: Supplementary file 7 — Additional file 7: Supplementary Figure 7. BSP expression after different treatments during 48 h in MCF-7. BSP was evidenced by immunofluorescence and is expressed in the cytoplasm. Estrogens (E) and CDCA (CDCA) induced an increase of BSP expression compared to the control (C). 4-hydroxytamoxifen (T), fulvestrant (F), LCA (L) and Z-guggulsterone (G) caused no variation in BSP expression compared to the control (C). 4-hydroxytamoxifen and fulvestrant in combined with estrogens or CDCA (E + T, E + F, CDCA+T, CDCA+F) caused a decrease of BSP expression compared to estrogens (E) or CDCA (CDCA). LCA and Z-guggulsterone in combined with CDCA (CDCA+L, CDCA+G) caused a decrease in BSP expression compared to CDCA (CDCA). Scale bars = 100 μm. [file 12885_2020_7106_MOESM7_ESM.pdf]

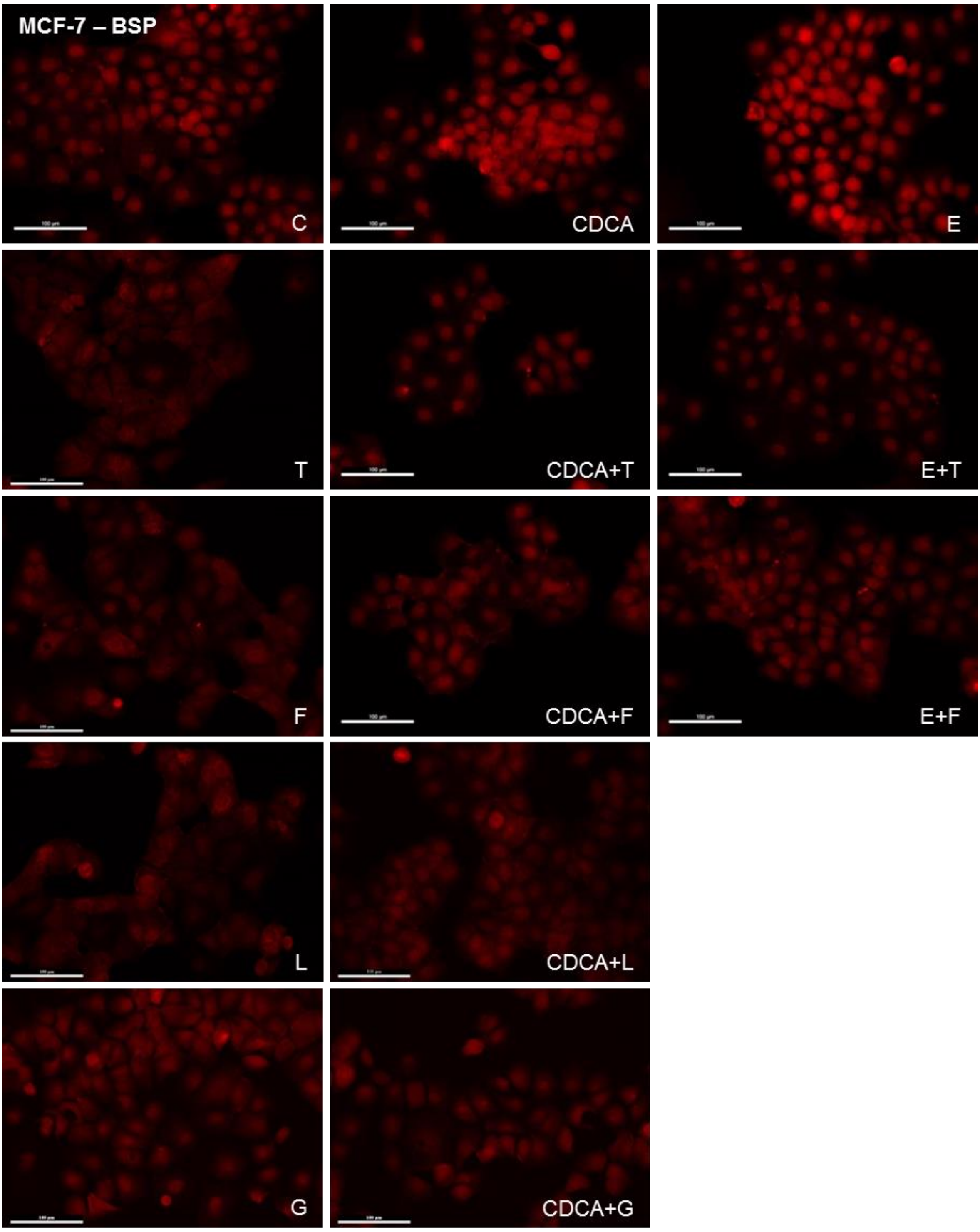

Supplementary Figure 7: BSP expression after different treatments during 48h in MCF-7. BSP was evidenced by immunofluorescence and is expressed in the cytoplasm. Estrogens (E) and CDCA (CDCA) induced an increase of BSP expression compared to the control (C). 4-hydroxytamoxifen (T), fulvestrant (F), LCA (L) and Z-guggulsterone (G) caused no variation in BSP expression compared to the control (C). 4-hydroxytamoxifen and fulvestrant in combined with estrogens or CDCA (E+T, E+F, CDCA+T, CDCA+F) caused a decrease of BSP expression compared to estrogens (E) or CDCA (CDCA). LCA and Z-guggulsterone in combined with CDCA (CDCA+L, CDCA+G) caused a decrease in BSP expression compared to CDCA (CDCA). Scale bars = 100 µm.
