# Supplementary material for: Farnesoid X receptor as marker of osteotropism of breast cancers through its role in the osteomimetism of tumor cells
Source: BMC Cancer. 2020 Jul 10;20:640. doi: 10.1186/s12885-020-07106-7 (PMC7350202; doi:10.1186/s12885-020-07106-7)
Supplement: Supplementary file 9 — Additional file 9: Supplementary Figure 9. Full-length Western Blotting with the cropped area corresponding to the image illustrated in Fig. 6e of the main text. Effect of FXR knock down on the synthesis of bone proteins. Scr: scramble, tested clones (4, 7, 8, 13, 15, A, B, C). A: Western blotting of FXR in MCF-7 cells after exposure to shRNA. Immuno bands are quantified and normalized with β-actin expression (illustrated in full blot B). Immunoreactive band intensities were quantified using the software ImageJ®. [file 12885_2020_7106_MOESM9_ESM.pdf]

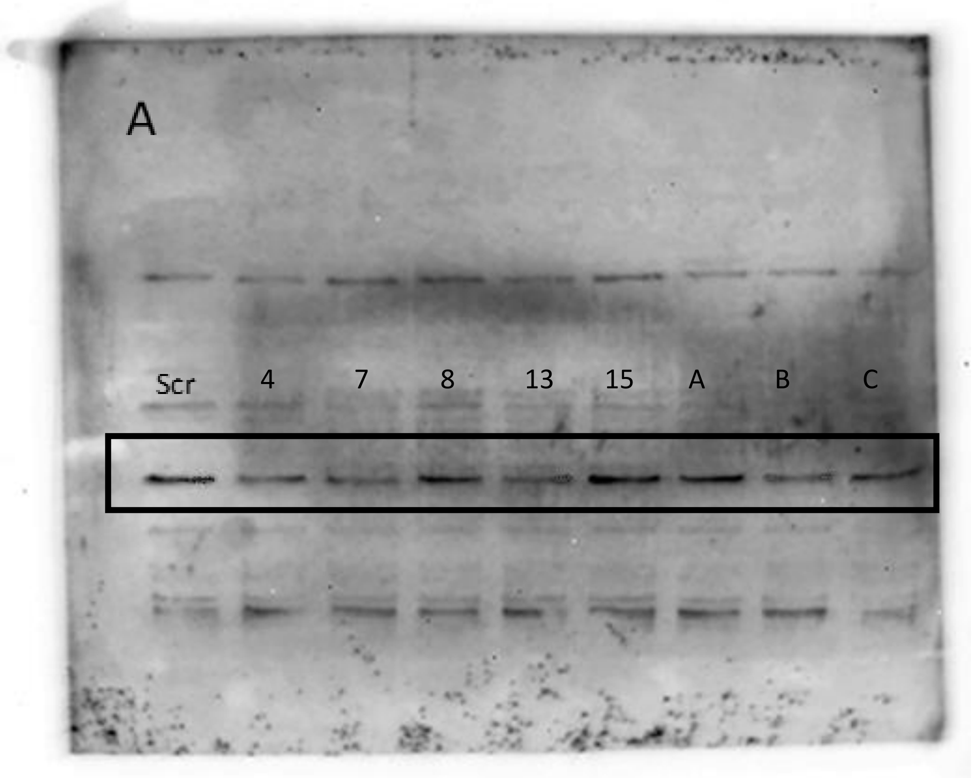

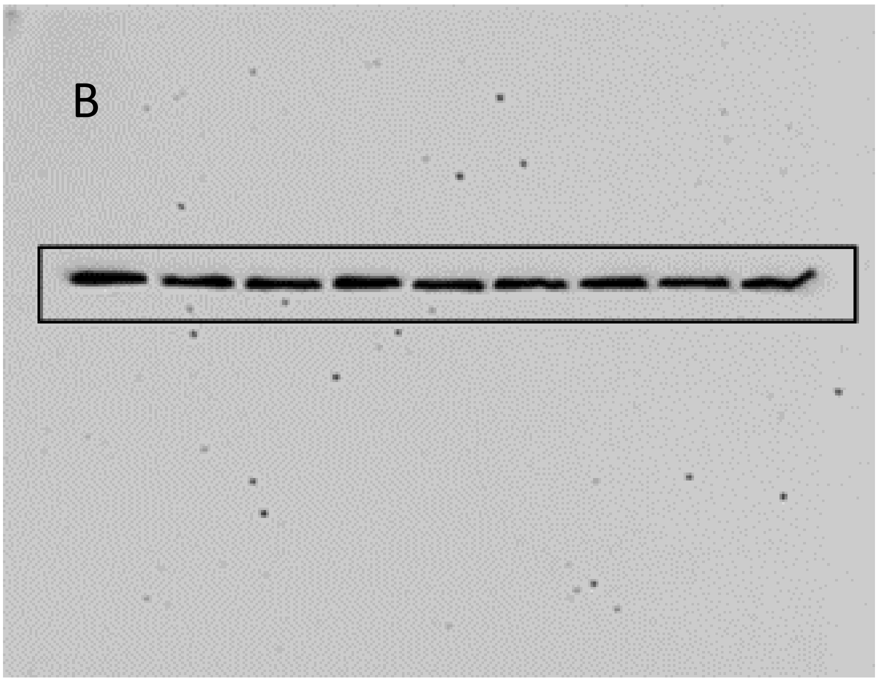

Supplementary figure 9 : Full-length Western Blotting with the cropped area corresponding to the image illustrated in figure 6E of the main text. Effect of FXR knock down on the synthesis of bone proteins. scr: scramble, tested clones (4, 7, 8, 13, 15, A, B, C). A: Western blotting of FXR in MCF-7 cells after exposure to shRNA. Immuno bands are quantified and normalized with β-actin expression (illustrated in full blot B). Immunoreactive band intensities were quantified using the software ImageJ®.
