# Supplementary material for: Farnesoid X receptor as marker of osteotropism of breast cancers through its role in the osteomimetism of tumor cells
Source: BMC Cancer. 2020 Jul 10;20:640. doi: 10.1186/s12885-020-07106-7 (PMC7350202; doi:10.1186/s12885-020-07106-7)
Supplement: Supplementary file 10 — Additional file 10. [file 12885_2020_7106_MOESM10_ESM.pdf]

Institut Jules Bordet

Association Hospitalière de Bruxelles
Centre des Tumeurs de l'ULB
Association régie par la loi du 8 juillet 1976
Rue Héger-Bordet 1 - 1000 Bruxelles
Tél.: (02) 541 31 11
Dexia 091-0097278-16

Comité d'Ethique

Tél : 02/541.35.95
Email secrétariat :
Comite.ethique@bordet.be

Président
Dr. Th. Gil

Vice-Président
Dr. D. Bron

Secrétaire
Dr. D. Lossignol

Membres
Dr. H. Bleiberg
Dr. J. Klastersky
Dr. M. Sosnowski
Dr. Th. Renard
M. P. Crombez
(suppléante : Mme B. Fernez)
Mme M. Colin
Mme M. Paesmans
Mme N. De Loore
Mme D. van Vyve
Mme S. Greco
Mme C. Deliens
Mme M. Nevraumont

LIST OF DOCUMENTS REVIEWED BY THE MEDICAL ETHICS COMMITTEE OF INSTITUT JULES BORDET - LEC

Réf. : as

Date : 10/09/2015

Intern number : 2437

Investigator : DR. F. JOURNE

Title

Rôle du FXR dans le cancer du sein

Including

Reply letter dd 31/08/2015
ISF (corrected)
Project description

LEADING ETHICS COMMITTEE'S DECISION

X Approval
☐ **Approval with conditions**
☐ **Need for extra information**
☐ **Disapproval**

Signature of the Chairman of the Central Ethics Committee

Name : Dr. Th. GIL

Date : 10/09/2015

List of members Ethics Committee (OM011)
(from 01/06/2015)

Meeting dd 10/09/2015

CE2437

			Attendance
DR. TH. GIL	Chairman	Physician	Yes
DR. D. BRON	Vice Chairman	Physician	No
DR. D. LOSSIGNOL	Secretary	Physician	Yes
DR. H. BLEIBERG	External member	Physician	No
DR. J. KLASTERSKY	Member	Physician	No
DR. M. SOSNOWSKI	Member	Physician	Yes
DR. TH. RENARD	External member	General Practitioner	Yes
P. CROMBEZ	Member	Head Nurse	Yes
B. FERNEZ	Member	Head Nurse	No
M. COLIN	External member	Nurse / Logopedist	Yes
M. PAESMANS	Member	Statistician	No
D. VAN VYVE	Member	Lawyer	Yes
S. GRECO	Member	Lawyer	No
N. DE LOORE	External member	Lay person	Yes
C. DELIENS	Member	Pharmacist	No
M. NEVRAUMONT	Member	Pharmacist	No

The Ethics Committee operates according to ICH Good Clinical Practice and local applicable regulations
